# Strategy for Identifying Dendritic Cell-Processed CD4^+^ T Cell Epitopes from the HIV Gag p24 Protein

**DOI:** 10.1371/journal.pone.0041897

**Published:** 2012-07-30

**Authors:** Leonia Bozzacco, Haiqiang Yu, Jörn Dengjel, Christine Trumpfheller, Henry A. Zebroski, Nawei Zhang, Victoria Küttner, Beatrix M. Ueberheide, Haiteng Deng, Brian T. Chait, Ralph M. Steinman, Svetlana Mojsov, David Fenyö

**Affiliations:** 1 Laboratory of Cellular Physiology and Immunology and Chris Browne Center, The Rockefeller University, New York, New York, United States of America; 2 Proteomics Resource Center, The Rockefeller University, New York, New York, United States of America; 3 Freiburg Institute for Advanced Studies, University of Freiburg, Freiburg, Germany; 4 Center for Biological Systems Analysis, University of Freiburg, Freiburg, Germany; 5 Laboratory of Mass Spectrometry and Gaseous Ion Chemistry, The Rockefeller University, New York, New York, United States of America; 6 Laboratory of Computational Proteomics, Center for Health Informatics and Bioinformatics, New York University Medical Center, New York, New York, United States of America; University of Bergen, Norway

## Abstract

Mass Spectrometry (MS) is becoming a preferred method to identify class I and class II peptides presented on major histocompability complexes (MHC) on antigen presenting cells (APC). We describe a combined computational and MS approach to identify exogenous MHC II peptides presented on mouse spleen dendritic cells (DCs). This approach enables rapid, effective screening of a large number of possible peptides by a computer-assisted strategy that utilizes the extraordinary human ability for pattern recognition. To test the efficacy of the approach, a mixture of epitope peptide mimics (mimetopes) from HIV gag p24 sequence were added exogenously to Fms-like tyrosine kinase 3 ligand (Flt3L)-mobilized splenic DCs. We identified the exogenously added peptide, VDRFYKTLRAEQASQ, and a second peptide, DRFYKLTRAEQASQ, derived from the original exogenously added 15-mer peptide. Furthermore, we demonstrated that our strategy works efficiently with HIV gag p24 protein when delivered, as vaccine protein, to Flt3L expanded mouse splenic DCs *in vitro* through the DEC-205 receptor. We found that the same MHC II-bound HIV gag p24 peptides, VDRFYKTLRAEQASQ and DRFYKLTRAEQASQ, were naturally processed from anti-DEC-205 HIV gag p24 protein and presented on DCs. The two identified VDRFYKTLRAEQASQ and DRFYKLTRAEQASQ MHC II-bound HIV gag p24 peptides elicited CD4^+^ T-cell mediated responses *in vitro*. Their presentation by DCs to antigen-specific T cells was inhibited by chloroquine (CQ), indicating that optimal presentation of these exogenously added peptides required uptake and vesicular trafficking in mature DCs. These results support the application of our strategy to identify and characterize peptide epitopes derived from vaccine proteins processed by DCs and thus has the potential to greatly accelerate DC-based vaccine development.

## Introduction

Cellular immune responses against any foreign non-self antigens depend upon recognition of peptides presented on the surface of antigen presenting cells (APCs), such as dendritic cells (DCs) [1]. Antigenic peptides are derived from the processing of exogenous proteins and are presented on MHC II molecules that are abundantly expressed on DCs [2]. Studies analyzing antigen presentation generally require the use of antigen-specific T cells isolated from immunized animals or human volunteers. Indeed, T cell epitopes are defined by screening sets of partially overlapping synthetic peptides covering the entire sequence of the known antigenic protein [3], or covering the entire proteome if no protein antigen is known. However, disadvantages of using T cell based-assays are the difficulty in quantitating peptides presented on the MHC complex by an absolute measurement and the large number of peptides that need to be assayed [4].

Other approaches for identification of T cell epitopes use predictive computational models of MHC binding affinity to obtain candidate peptides [5]. To date many epitopes have been identified using HLA binding motifs and peptide prediction algorithms. For example, Assarsson et al. [6] identified 54 novel epitopes from more than 4000 computer-defined peptides with HLA binding motifs, including 38 that were CD8^+^ T cell specific. However, it is not known whether these are immunodominant or subdominant within any given CD8^+^ T cell response.

Mass spectrometry (MS) provides an effective means to sample and quantitate MHC II-associated self- peptides from DCs for many MHC II proteins [7], [8]. Here we seek to identify among the total mixture of MHC II peptides the specific antigenic peptides of low abundance derived from exogenous proteins. Due to the limited dynamic range of MS instrumentation, typically only a small fraction of the observed peptides in a complex sample is successfully sequenced, even with the fastest current instruments [9]. Targeted data acquisition approaches have been shown to achieve much lower detection limits [10], but only a limited number of peptides can be targeted in any given analysis. Thus, current data-dependent analysis will not consistently capture the peptides of interest, and targeted data acquisition cannot effectively cover the large number of possibilities.

**Figure 1:**
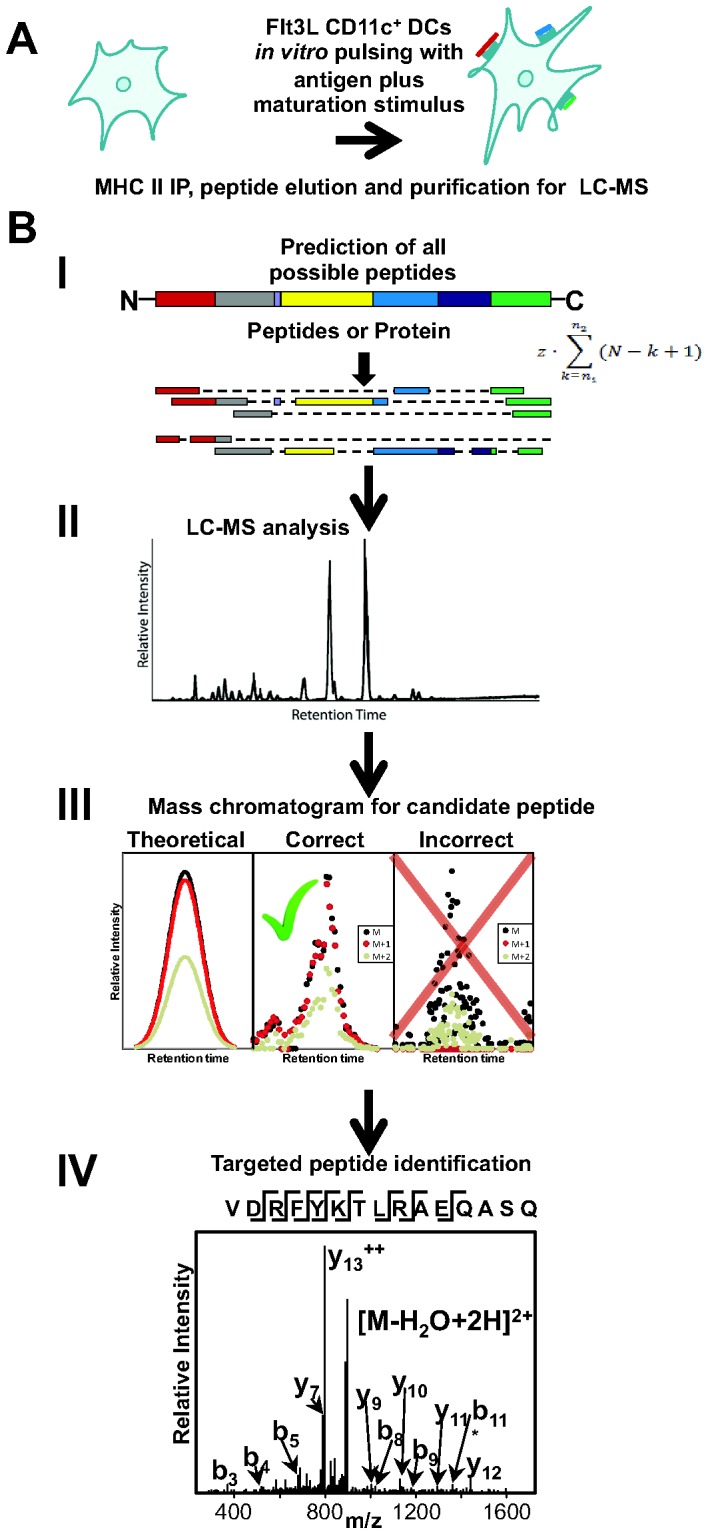
A schematic overview of the strategy to identify and analyze MHC II peptides presented by DCs from exogenous protein and peptide sequences. (A) MHC II peptide-complexes are isolated by immunoprecipitation from DCs pulsed *in vitro* with antigen. (B, I) A list of all possible theoretical peptides is generated from the amino acid sequence of the known antigen. (B, II) The masses of the enriched DC MHC II peptides are measured by LC-MS analysis. (B, III) The mass chromatograms for the isotopic peaks of all possible theoretical peptides are visualized as color-coded plots. (B, IV) The observed peptide masses are selected for targeted MS/MS analysis.

Here, we solved this problem by a four step-approach (Figure 1B). First, we enumerate all theoretical possible MHC-II peptides from the exogenously added protein sequence. Second, we obtain mass spectra of the enriched MHC II peptides from DCs. Third, we search these spectra for mass evidence of the theoretical peptides. Fourth, if a potential match is found, this is tested by targeted tandem mass spectrometry.

To evaluate this strategy we chose HIV gag p24 peptide sequences because the gag protein plays an important role in inducing protective T cell immunity to HIV [11] and is consequently a component of several experimental vaccines [3], [12], [13], [14]. Indeed, identification of the CD4^+^ T cell epitopes presented on class II MHC complexes are of particular interest because of their role in the development of protective antibody responses induced by vaccination [15]. We used our strategy to identify two MHC II-bound HIV gag p24 peptides, which elicited strong T cell responses *in vitro*. We also demonstrate that presentation of MHC II-associated HIV gag peptides by mature DCs takes place intracellularly.

## Results

### EpiSifter for Rapid Detection of Specific MHC II Peptides

To detect, identify and measure exogenous MHC II-bound peptides presented by DCs among the abundant and complex mixture of endogenous peptides, Flt3L-mobilized DCs are pulsed *in vitro* with synthetic peptides or with a protein [1], [7], [16], [17]. DC MHC II-complexes are then enriched by immunoprecipitation, whereupon bound peptides are eluted and purified for MS analysis (Figure 1A) [7]. However, it is difficult to detect these specific low abundance peptides by conventional data-dependent acquisition. In data-dependent acquisition, the masses of all peptides eluting at a particular time are first measured. Then, based on this measurement, starting typically from the highest intensity peak species, a number of peptide ions are sequentially isolated and their fragmentation spectra (MS/MS) acquired. In highly complex samples, as is the case for DC MHC II peptide mixture, this method often fails to detect the less abundant species of interest [18].

To overcome this limitation we developed a four step strategy (Figure 1B): (I) the program EpiSifter generates all theoretical possible MHC II peptides, ranging from 9 up to 25 amino acid in length from the amino acid sequence of the known antigen (peptides or protein). Their possible mass to charge (m/z) ratios are calculated along with their isotope ratios. Typical modifications, like oxidized methionine, are also considered for calculation of the theoretical m/z values. (II) The masses of the enriched MHC II peptides isolated from DCs are measured with high accuracy by LC-MS analysis. (III) Mass chromatograms of the m/z ratios for all the theoretically possible peptides are visualized as color-coded plots, allowing a fast and efficient checking of the spectra. (IV) Peptides, where signals are observed at the correct m/z ratio, have the expected chromatographic peak width and have the correct isotope distribution, are selected for targeted MS/MS analysis in a separate run to test their sequence and identity.

Using this strategy we can rapidly find mass evidence for putative exogenous MHC II peptides and then test their identity by targeted MS/MS. An additional advantage of this strategy is that it identifies the most abundant charge state for subsequent targeted analysis, further reducing the list of candidate masses dramatically.

### Identification of DC MHC II-bound Peptides from an Exogenous HIV gag p24 Peptide Mixture

To test the strategy, DCs were loaded with four HIV gag p24 15-mer peptides at concentrations of 1 µM each. These were previously identified by ELISPOT assay as mimetopes after anti-DEC gag p24 antibody vaccine immunization in two distinct mouse haplotypes (Table 1) [3]. EpiSifter generated a list of all 321 theoretically possible peptides, 9 to 15 amino acids in length (Table S1), for the four HIV gag 15 mer mimetope sequences by applying the following rules: a) removing up to four amino acids from both termini; b) allowing charge states from +1 to +4; c) considering methionine in its oxidized and unoxidized form.

**Table 1:** HIV gag p24 immunoreactive peptides previously identified by ELISPOT assay.

BALB/c(I-A/E^d^)	A9	aa 165–179	SPEVIPMFSALSEGA
	C8	aa 257–271	PVGEIYKRWIILGLN
C57BL/6 (I-A^b^)	A4	aa 145–159	QAISPRTLNAWVKVV
	D6	aa 297–311	VDRFYKTLRAEQASQ

HIV gag p24 mimetope peptides identified previously from 15-mer peptide pools to stimulate IFNγ secretion by CD4^+^ T cells in two MHC haplotypes after anti-DEC-HIV gag p24 immunization [3].

**Figure 2:**
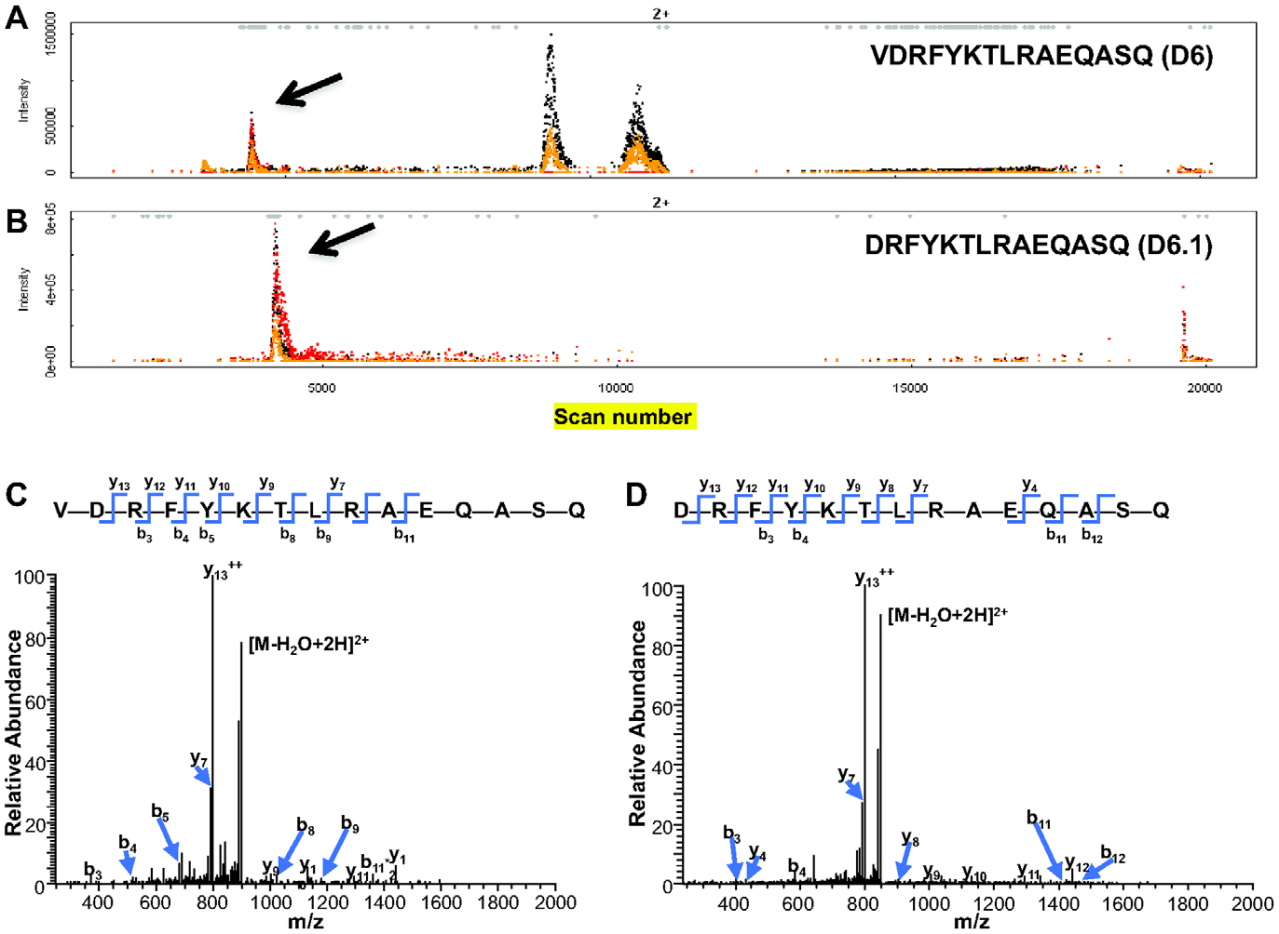
Query of DC MHC II- bound HIV gag p24 peptides derived from an exogenous peptide mixture. Full scan profile of two predicted peptides generated by EpiSifter. The black histogram indicates the monoisotopic peak, the red one corresponds to the first ^13^C isotope, while the grey-coded one represents the second. The isotope envelopes marked with arrows correspond to the peptide sequences: (A) VDRFYKTLRAEQASQ (designated as D6) and (B) DRFYKTLRAEQASQ (designated as D6.1). (C, D) MS/MS spectra of the query peptides, VDRFYKTLRAEQASQ (m/z 906.48, z = 2) (C) and DRFYKTLRAEQASQ (m/z 856.94, z = 2) (D).

Following LC-MS analysis of DC MHC II-bound peptides [7], the mass chromatograms were visualized with EpiSifter for each theoretically predicted peptide (Table S1). Figure 2 and Figure S1 show the mass chromatograms for DC MHC II-bound HIV gag p24 peptides from one representative experiment of three biological replicates. By manual inspection of the 321 mass chromatograms (one for each predicted peptide) we observed for two peptide sequences, VDRFYKTLRAQASQ (D6 in Table 1) and DRFYKTLRAQASQ (designated D6.1), a region in the mass chromatogram where the first three isotopes of the peptide were present at approximately the expected intensity ratios (Figure 2A and 2B) [19]. There was no evidence in the mass chromatograms for the other possible versions of the other three HIV gag p24 peptides added to DCs (Figure S1A, S1B, S1C). By conventional database search of the original data, MHC II HIV gag p24 peptides could not be identified among the endogenous mouse MHC II peptides. (Table S2) [7]. Next, we performed targeted MS/MS analysis for the two peptides shown in Figure 2A and 2B. Figure 2C and 2D show the MS/MS spectra of the double charged peptides VDRFYKTLRAQASQ and DRFYKTLRAQASQ at m/z 906.48 and m/z 856.94 respectively. The corresponding MS/MS spectra of MHC II-bound HIV gag p24 peptide sequences (Figure 2C and 2D) were identical to the ion product spectra of the synthetic isotope labeled peptides VDRFYKT*LRAQASQ (m/z 909.99, z = 2, *^13^C_6_N_1_ **L**) and DRFYKT*LRAQASQ (m/z 860.45, z = 2, *^13^C_6_N_1_ **L**) respectively (Table S3), confirming unambiguously the validity of the identification (Figure S2).

We concluded that by using EpiSifter strategy we quickly and successfully identified the exogenously added peptide, VDRFYKTLRAEQASQ, and a second peptide, DRFYKLTRAEQASQ, derived from the original added HIV gag p24 15-mer peptide by cleavage of the N-terminal amino acid (Table 1) (see below).

### The Two MHC II-bound HIV gag p24 Peptides Presented by DCs are Derived from a Single Exogenous Peptide Sequence

**Figure 3:**
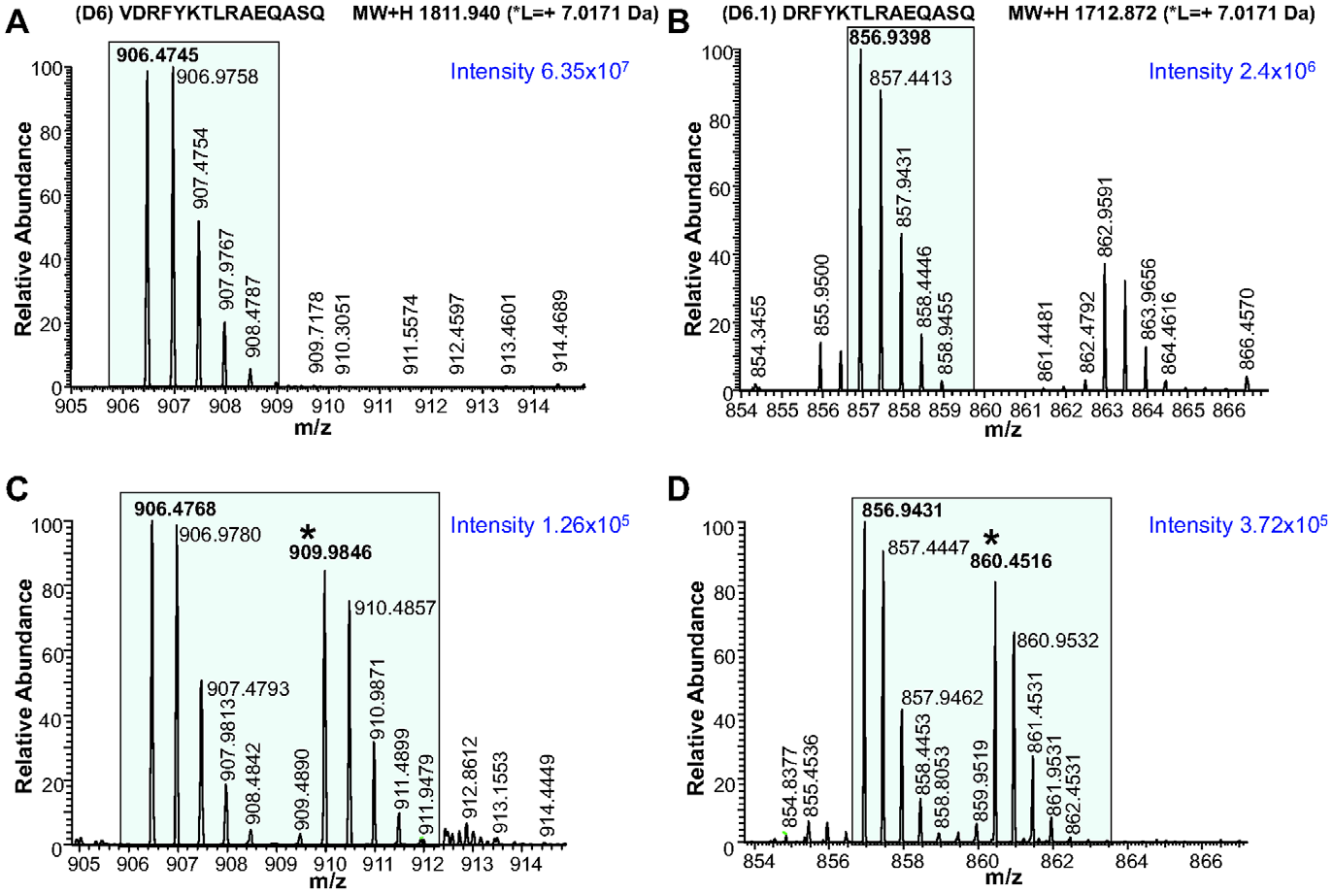
Detection of VDRFYKTLRAEQASQ and DRFYKTLRAEQASQ as DC MHC II-bound peptides after DCs were pulsed *in vitro* with VDRFYKTLRAEQASQ HIV gag p24 peptide sequence. (A) MS profile of the VDRFYKTLRAEQASQ peptide identified in the MHC II peptide mixture eluted from one representative sample. (B) MS profile of the DRFYKTLRAEQASQ identified from the same sample as in A. (C) MS profile of the VDRFYKTLRAEQASQ isotope pair eluted from DCs pulsed with an equimolar ratio of heavy and light VDRFYKTLRAEQASQ peptide (Table S3). (D) MS profile of the DRFYKTLRAEQASQ peptide pair identified from the same sample as in (C). Isotope peaks corresponding to m/z (z = 2) values for the detected peptides are shown in the shaded area. Heavy isotope peaks are marked with a (*).

To test that the DRFYKLTRAEQASQ peptide is indeed derived from the original exogenously added VDRFYKLTRAEQASQ peptide sequence by an active physiological process, we performed a set of experiments, the results of which are provided in Figure 3. Figures 3A and 3B show details of the mass spectra of VDRFYKTLRAQASQ (m/z 906.48, z = 2) and DRFYKTLRAQASQ (m/z 856.94, z = 2) recorded from the MHC II eluted peptide mixture. Figure 3C and 3D show mass spectra of the isotope pairs corresponding to VDRFYKTLRAQASQ (Figure 3C) and DRFYKTLRAQASQ (Figure 3D) peptides when DCs were pulsed with an equimolar mixture of heavy and light isotope forms of HIV gag p24 VDRFYKTLRAQASQ peptide (Table S3). Observation of these pairs supports the hypothesis that the 14-mer peptide DRFYKTLRAQASQ is generated from the original 15-mer peptide sequence VDRFYKTLRAQASQ added to DCs *in vitro*.

To examine in more detail the processing of the exogenously added peptides by DCs, we repeated the same experiment in the presence of 20 mM ammonium chloride (NH_4_Cl) as an inhibitor of endo-lysosome compartment acidification. In the presence of 20 mM NH_4_Cl, we did not detect any signal at the expected m/z values (Figure S3A and S3B), suggesting that binding to MHC II complexes of the HIV gag p24 peptides VDRFYKTLRAQASQ and DRFYKTLRAQASQ occurred intracellularly and was inhibited by 20 mM NH_4_Cl.

### Exogenously Added HIV gag p24 Peptides are Presented on DC MHC II Molecules in a Similar Concentration Range as the Endogenous Self-MHC II Peptides

To evaluate the absolute abundance of MHC II-bound HIV gag VDRFYKTLRAQASQ and DRFYKTLRAQASQ peptides presented on DCs, we performed quantitative analysis by using isotope labeled peptide standards [7]. Figure S4 shows the mass chromatogram of the VDRFYKTLRAQASQ and the DRFYKTLRAQASQ peptide pairs measured in one representative experiment. We extrapolated from three biological replicates that the amount of VDRFYKTLRAQASQ presented on MHC II molecules of DCs was 19±4 fmol/µL, corresponding to 590±120 copies per cell. Quantitation of DRFYKTLRAQASQ peptide showed that it was presented on DC MHC II molecules at 1800±120 copies per cell, calculated from 60±4 fmol/µL.

In conclusion, we observed that DCs presented exogenous HIV gag p24 peptides on MHC II complexes in the range of concentration detected previously for the endogenous MHC II peptide repertoire, i.e. 2.5 fmol/µL to 12 pmol/µL or from approximately 13 to 2×10^5^ copies per DC [7].

### Identification of VDRFYKTLRAEQASQ and DRFYKTLRAEQASQ as MHC II-bound Peptides Derived from Processing of Anti-DEC 205-HIV gag p24 Protein by DCs

To extend our strategy to a relevant vaccine protein delivered as exogenous antigen, we used anti-DEC 205 HIV gag p24 protein, delivered specifically to DCs *in vitro* through the cell-specific DEC-205 receptor [3], [12], [13], [14]. We previously demonstrated that introduction of a protein within anti-DEC-205 antibody greatly enhances the capacity of Flt3L DCs to present an antigen, such as for example HIV gag p24 protein [1], [7], [16], [17]. Here, we first pulsed mouse spleen Flt3L-DCs *in vitro* for 5 h with 1 µg/mL (5 pM) of anti-DEC-205 HIV gag p24 protein and then we eluted MHC II peptides as described above. In this case, EpiSifter generated a list of all 8189 theoretically possible peptides, 7–27 amino acids in length (Table S4), for the HIV gag p24 protein sequence (aa 133–363 derived from HIV isolate BH10), considering charge states from +1 to +4 and methionine oxidation.

**Figure 4:**
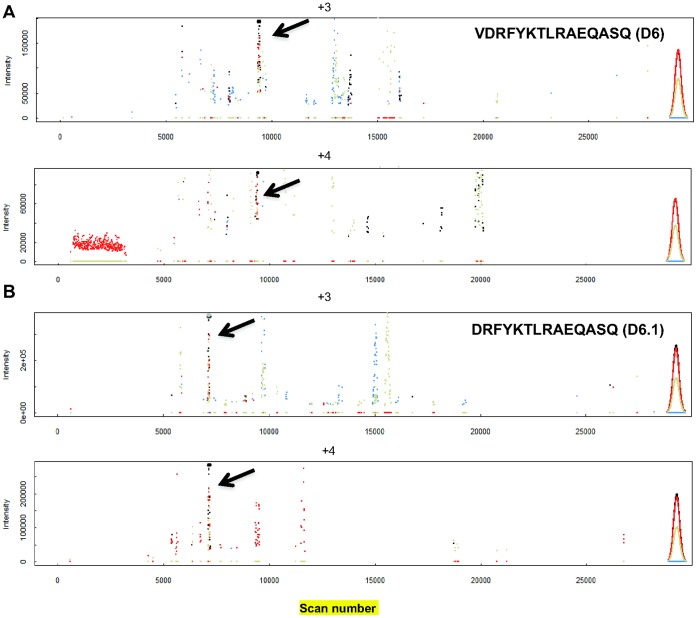
Query of DC MHC II- bound peptides derived from exogenously added anti-DEC-205 HIV gag p24 protein. (A, B) Full scan profile of VDRFYKTLRAEQASQ (D6) and DRFYKTLRAEQASQ (D6.1) peptides generated by EpiSifter. The isotope envelopes marked with arrows correspond to the previously identified peptide sequences: (A) VDRFYKTLRAEQASQ (D6) and (B) DRFYKTLRAEQASQ (D6.1), both found in the +3 and +4 charge states.

Following LC-MS analysis of eluted DC MHC II-bound peptides, the mass chromatograms were visualized with EpiSifter for all the theoretically predicted peptides. Figure 4 shows the mass chromatograms of the previously identified (Figure 2) HIV gag p24 peptides VDRFYKTLRAEQASQ (D6) and DRFYKTLRAEQASQ (D6.1), as DC MHC II-bound peptides naturally processed from the anti-DEC-205 HIV gag p24 protein added exogenously to splenic Flt3L-DCs *in vitro*. We observed for the two peptides, VDRFYKTLRAEQASQ (D6) and DRFYKTLRAEQASQ (D6.1) identified above, a region in the mass chromatogram where the first three isotopic peaks of the peptides were present with similar predicted intensities, in two different charge states, +3 and +4, (Figure 4A and 4B). By EpiSifter we did not detect the other previously described HIV gag p24 mimetopes (Table 1), in agreement with our initial observations with exogenously added 15-mer peptides (Figure S1).

**Figure 5:**
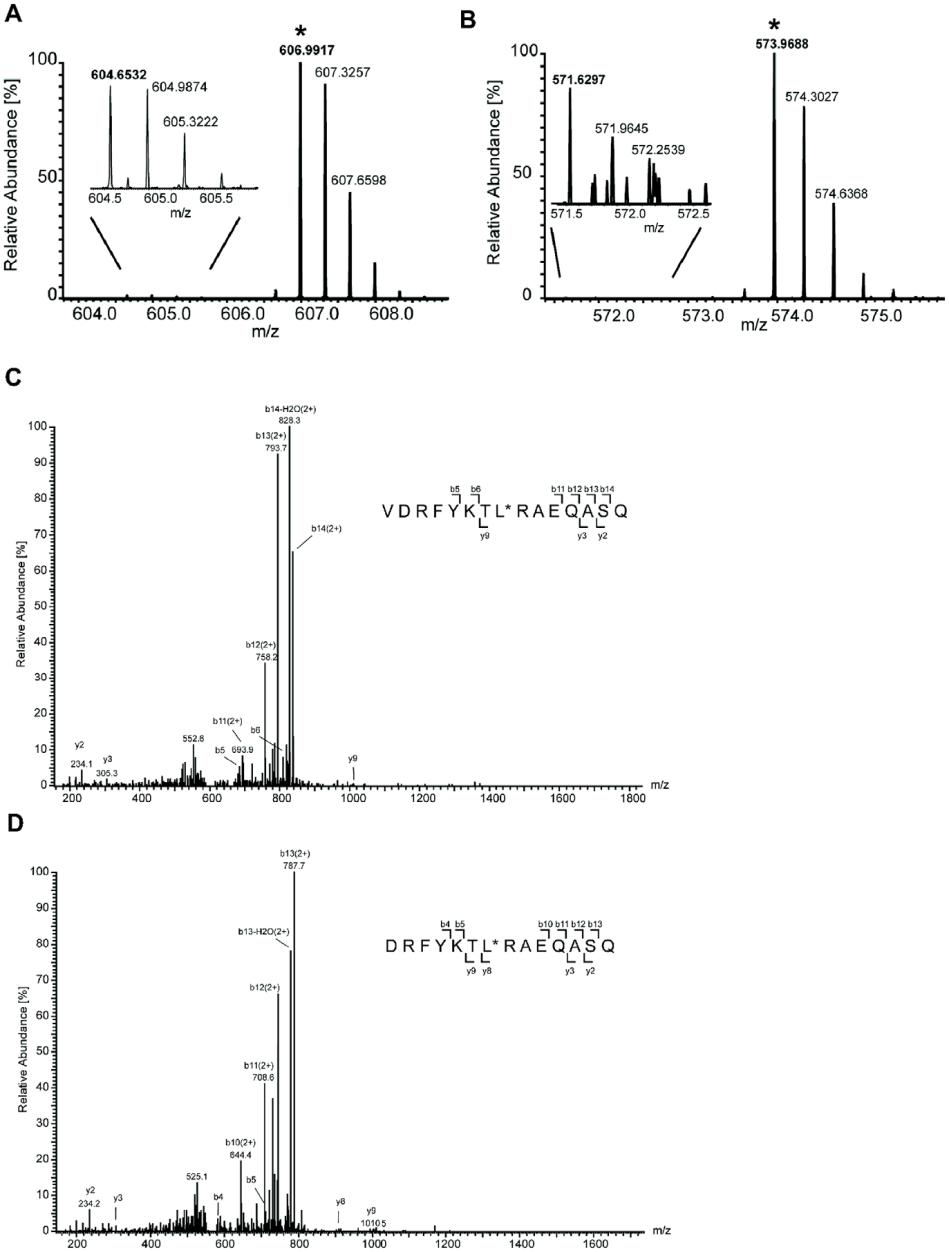
Detection of VDRFYKTLRAEQASQ (D6) and DRFYKTLRAEQASQ (D6.1) as DC MHC II-bound peptides after DCs were pulsed *in vitro* with anti-DEC-205 HIV gag p24 protein. One representative experiment of two biological replicates is shown. MS profile of VDRFYKTLRAEQASQ (D6) (A) and DRFYKTLRAEQASQ (D6.1) (B) isotope peptide pair identified as MHC II-bound peptides derived from anti-DEC-205 HIV gag p24 protein, using synthetic isotopically labeled peptides as internal standards, marked with (*). For clarity, the restricted mass range of interest was zoomed in. (C, D) Identification of MHC II-bound peptides from anti-DEC-205 HIV gag p24 protein. MS/MS spectra were obtained for the synthetic isotope labeled peptides at m/z 606.9917, z = 3 for VDRFYKT*LRAQASQ (C) and at m/z 573.9688, z = 3 for DRFYKT*LRAQASQ (D) recorded in the LC-MS analysis shown in Figure 5A and 5B. The isotopically labeled amino acid is labeled with a (*). The corresponding y and b series are marked.

We performed MS/MS analysis on the experiment shown in Figure 5 but we did not obtain the sequences of VDRFYKTLRAQASQ and DRFYKTLRAQASQ peptides. We hypothesized that the sequencing could not be performed because they were present in low amounts, thus preventing successful fragmentation of the peptide ions. Then, to prove the identity of VDRFYKTLRAQASQ and DRFYKTLRAQASQ peptides, we introduced their corresponding synthetic isotopically labeled peptides VDRFYKT*LRAQASQ (*^13^C_6_N_1_ **L**) and DRFYKT*LRAQASQ (*^13^C_6_N_1_ **L**) (Table S3) into the sample prior to LC-MS run. This pair of peptides (light and heavy isotopes) is chemically identical, with the same amino acid sequence and easily detectable because it coelutes during LC separation. In Figure 5 we show the chromatographic profile of a representative example of LC-MS experiment for VDRFYKTLRAEQASQ (Figure 5A) and DRFYKTLRAEQASQ (Figure 5B) isotope peptide pair. We enlarged the area of the spectrum comprising the masses of interest to show the extracted ion chromatographic profile of the triple charged peptides VDRFYKTLRAEQASQ (Figure 5A) and DRFYKTLRAEQASQ (Figure 5B) at m/z 604.6532 (z = 3) and m/z 571.6297 (z = 3), respectively. The simultaneous detection of peaks at m/z 604.6532 and 606.9917 (z = 3, Figure 5A) indicated the co-elution of VDRFYKTLRAQASQ and VDRFYKT*LRAQASQ peptides during LC separation (retention time  = 50.19 minute, data not shown). We calculated as 2.1 and 1.98 ppm the mass deviation between the measured m/z values (604.6532 and 606.9917 for VDRFYKTLRAEQASQ and VDRFYKT*LRAQASQ, respectively) and the theoretical values, 604.6519 and 606.9905, of the VDRFYKTLRAEQASQ peptide pair. In other words, the exact mass measurement of the VDRFYKTLRAEQASQ peptide pair and co-elution during LC separation led to the unambiguous peptide assignment.

The same method was applied to confirm the presence of peptide DRFYKTLRAQASQ in the sample. The measured m/z of DRFYKTLRAEQASQ and DRFYKT*LRAQASQ (*^13^C_6_N_1_ **L**) peptides were 571.6297 and 573.9688 respectively with only 1.75 ppm and 1.91 ppm mass deviation (Figure 5 B), compared to the theoretical mass, 571.6287 and 573.9677, calculated respectively for DRFYKTLRAEQASQ and DRFYKT*LRAQASQ peptide sequences.

Last, the MS/MS spectra obtained for the synthetic isotope labeled peptide VDRFYKT*LRAQASQ at m/z 606.9917, z = 3 and for DRFYKT*LRAQASQ at m/z 573.9688, z = 3 are shown in Figure 5C and 5D, respectively. Their MS/MS sequences further confirmed the validity of the identification of VDRFYKTLRAEQASQ at m/z 604.6532 (Figure5A and 5C) and DRFYKTLRAEQASQ at m/z 571.6297 (Figure 5B and 5D) as DC MHC II-bound peptides derived from anti-DEC-205 HIV gag p24 protein exogenously added to mouse splenic DCs. Taken together the above data provide strong support for the presence of naturally processed VDRFYKTLRAEQASQ (D6) and DRFYKTLRAEQASQ (D6.1) MHC II-associated peptides derived from anti-DEC 205 HIV gag p24 protein.

We concluded that by using EpiSifter strategy we quickly and successfully identified two peptides, VDRFYKTLRAEQASQ and DRFYKLTRAEQASQ as naturally processed DC MHC II-bound peptides derived from the exogenously added anti-DEC-205 HIV gag p24 protein. In addition, these data validated our findings with exogenously added VDRFYKTLRAEQASQ (D6) HIV gag p24 peptide identified in the first part of our work (Figure 2).

### DCs Display Low Amounts of Processed MHC II Peptides from Anti-DEC-205 HIV gag p24 Protein

We extracted quantitative data for the HIV gag p24 VDRFYKTLRAEQASQ (D6) and DRFYKLTRAEQASQ (D6.1) peptides derived from anti-DEC-205 HIV gag p24 protein in the experiment described in Figure 5. By using a known amount (1 ng) of isotopically labeled peptides standards [7], from a single quantitation analysis (Figure 5), we measured 1.4 fmol/µL of VDRFYKTLRAEQASQ (D6) and 0.14 fmol/µL of DRFYKTLRAEQASQ (D6.1), corresponding approximately to 17 and 1.6 copies/cell, respectively. As a control in the same experiment, we quantitated the amount of KELEEQLGPVAEETR MHC II peptide derived from endogenous apolipoprotein E [7], to be 180 copies/cell (15 fmol/µL) (Figure S5). This measurement is in good agreement with our previous quantitation of 548 copies per cell (42 fmol/µL) [7]. It falls within the 30% range of variability tolerated when different biological samples and different analytical runs are compared [20]. However, the values obtained for the VDRFYKTLRAEQASQ (D6) and of DRFYKTLRAEQASQ (D6.1) peptides presented on MHC II molecules on DCs may be an underestimation due to large differences in the peak intensities between the MHC II-bound peptides and the isotopically labeled standards added in the sample prior to the MS run.

### Ag-specific CD4^+^ T Cells Recognize MHC II-bound HIV gag Peptides Identified by LC-MS/MS

**Figure 6:**
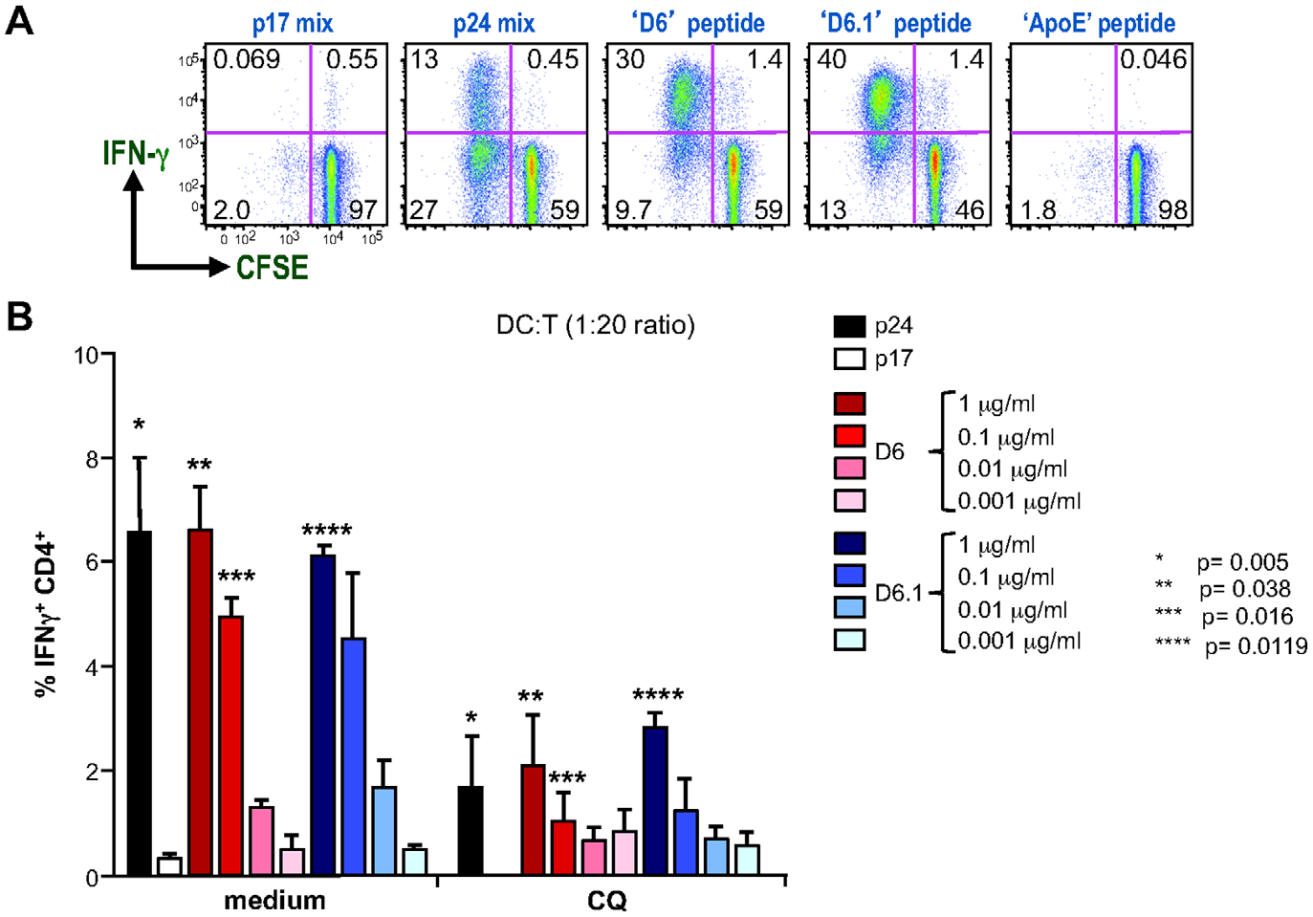
Immuno-reactivity of the MHC II-bound HIV gag p24 peptides measured by FACS analysis. (A) CFSE-labeled bulk splenocytes from HIV gag p24 immunized mice were stimulated with negative control HIV gag p17 peptide mix, positive control HIV gag p24 peptide mix and HIV gag VDRFYKTLRAEQASQ (D6), DRFYKTLRAEQASQ (D6.1) peptides and unrelated ApoE peptide (Figure S5). (B) Antigen presentation assay of MHC II–bound HIV gag p24 peptides in DC:T cell cocultures. Untreated (medium) and CQ-treated DCs were loaded with graded doses of peptides and added to CFSE-labeled purified T cells. Values represent mean ± SD of the percentage of CD4^+^ IFNγ^+^ T cells pooled from four independent experiments. Paired Student’s t-test was performed for untreated- *versus* CQ-treated DCs.

To determine the reactivity of VDRFYKTLRAQASQ and DRFYKTLRAQASQ MHC II- bound HIV gag p24 peptides identified with the present strategy, we performed 6-carboxyfluorescein succinimidyl ester (CFSE) T cell proliferation assays. Synthetic VDRFYKTLRAQASQ and DRFYKTLRAQASQ HIV gag p24 peptides were added to bulk splenocytes isolated from HIV gag p24 immunized mice [21]. In Figure 6A, we measured proliferation and IFNγ production in the presence of HIV gag p24 VDRFYKTLRAQASQ (D6) and DRFYKTLRAQASQ (D6.1) peptides. We observed that CD4^+^ T cells proliferated actively after restimulation with VDRFYKTLRAQASQ and DRFYKTLRAQASQ peptides as well as in presence of the 56-peptide mixture spanning the entire HIV gag p24 sequence (Figure 6A). No reactivity was measured with the negative control HIV gag p17 peptide mix [3] and with the endogenous MHC II apolipoprotein E (ApoE) peptide, which sequence is shown in Figure S5 [7].

We also compared the reactivity of VDRFYKTLRAQASQ peptide with the other three HIV gag p24 mimetopes (Table 1), which were not detected by LC-MS as MHC II-bound peptides (Figure S1). Only the HIV gag p24 VDRFYKTLRAQASQ peptide stimulated efficiently proliferation and IFNγ secretion of HIV p24 specific CD4^+^ T cells, comparably to the response with the 56-peptide mix HIV gag p24 (Figure S6). None of the other HIV gag p24 mimetopes were reactive in expanding antigen-specific bulk splenocytes.

Next, to directly assess the function of DCs in presenting the above-identified HIV gag p24 VDRFYKTLRAQASQ (D6) and DRFYKTLRAQASQ (D6.1) peptides to T cells, peptide pulsed DCs were cocultured with a fixed number of T cells isolated from HIV gag p24 immunized mice [21]. T cell proliferation and IFNγ secretion were measured as above. We used a T cell: DC ratio of 20∶1, at which only minimal level of unspecific T cell proliferation was detected. We observed a robust proliferation and cytokine secretion of CD4^+^ T cells when DCs were pulsed with graded dose of VDRFYKTLRAEQASQ (D6) and DRFYKTLRAEQASQ (D6.1) peptides (Figure 6B, left “medium” chart). And as expected no T cell activation was induced when DCs were pulsed with unspecific peptides (HIV gag p17).

Several reports have suggested that binding of exogenous mimetope peptides to MHC molecules may occur at the cell surface, without the need for intracellular processing [22]. Other reports have indicated that binding of exogenous peptides may take place intracellularly upon uptake of the peptides [23]. To begin to understand the mechanism of processing and presentation of exogenous peptides by DCs, we inhibited endosomal-lysosomal system acidification with chloroquine (CQ). In control experiments we first determined the optimal concentrations of CQ by using DC viability as an endpoint. Treatment of DCs with 100 µM CQ did not alter cell viability compared to untreated cells (1.8×10^6^ and 1.7×10^6^, respectively, recovered from initial 2×10^6^ DC/well). Since treatment with CQ distinctly inhibited T cell-activation by DCs in response to HIV gag p24 peptides, we inferred that intracellular processing of exogenously added VDRFYKTLRAQASQ was required, (Figure 6B, right “CQ” chart). In four independent experiments CD4^+^ T cell activation to peptide-loaded DCs was decreased by a median of 26% for VDRFYKTLRAQASQ (p = 0.038 compared to peptide alone at 1 µg/ml) and 46% for DRFYKTLRAQASQ peptide (p = 0.012 compared to peptide alone at 1 µg/ml). In line with other reports [24], [25], we found that inhibition of peptide presentation by NH_4_Cl was reversible after removal of NH_4_Cl by extensive washing of DCs with PBS (Figure S7). All together these results demonstrate that low concentrations of MHC II-associated HIV gag p24 VDRFYKTLRAQASQ and DRFYKTLRAQASQ peptides induce expansion of antigen-specific CD4^+^ T cells and that MHC II presentation of 15- or 14-mer peptides by mature DCs occurs through intracellular pathways.

Finally, we assessed the functional avidity of T cell responses following VDRFYKTLRAEQASQ and DRFYKTLRAEQASQ peptides presentation by DCs [26]. The magnitude of the CD4^+^ T cell response measured for each peptide concentration from Figure 6B was expressed as a percentage of the maximum response observed with 1×10^−6^ M peptide (Figure S8). The EC_50,_ calculated as estimation of functional avidity indicated that VDRFYKTLRAEQASQ and DRFYKTLRAEQASQ peptides, presented on MHC II molecules of DCs, have similar sensitivity for antigen-specific T cells (EC_50_ 3.7 and 3.6×10^−8^ M, respectively). For comparison, a 30 fold higher concentration of Her2 derived peptide, PDSLRDLSVF, amino acid 420–429, from the Her2/Neu breast cancer antigen [27] is required to induce antigen-specific T cell responses in a vaccine model for breast cancer [28]. The measured high avidity of T cells specific to HIV gag p24 VDRFYKTLRAEQASQ and DRFYKTLRAEQASQ peptides reinforces the use of the HIV gag p24 protein as a vaccine target in clinical studies.

## Discussion

We have described a strategy, which combines the use of a computational tool, EpiSifter, with LC-MS analysis, to identify antigen-specific MHC II peptides eluted from DCs. To test the strategy, we first used DCs that were pulsed *in vitro* with HIV gag p24 peptides and we identified two MHC II-bound HIV gag p24 sequences, VDRFYKTLRAQASQ and DRFYKTLRAQASQ, present at concentrations within the range of previously identified MHC II self-peptides on DCs [7]. For the reasons we discussed earlier, traditional data-dependent LC-MS/MS analysis was unable to identify (Table S2), these sequences and this limitation was overcome by the present strategy (Figure 1B).

There have been only few reports of exogenous MHC-bound peptides identified by LC-MS/MS analysis, and only following exhaustive manual interrogation of thousands of MS/MS spectra or by prior peptide fractionation of complex mixture followed by screening of antigenic peptide fractions in T cell assays [4], [29], [30], [31], [32]. The benefit of our strategy is that it depends in the first instance on the acquisition of accurate mass measurements that narrow the potential m/z values, which are compatible with predicted peptides. The EpiSifter predicts all possible candidate peptides and uses an effective graph representation to target only those peptide masses that are actually detected. Then, targeted MS sequencing is performed for the candidate peptides that are initially best revealed by the program (Figure 1B). This strategy is particularly useful for the analysis of unanticipated peptide species where other techniques such as selected reaction monitoring (SRM) or targeted analyses are not feasible.

In the initial experiments (Figure 2) we used VDRFYKTLRAQASQ (D6) and other three HIV gag p24 mimetope sequences (Table 1) as a proof of concept. However, this strategy is applicable to any other protein sequence. Indeed, we extended our approach and evaluated antigen processing following exogenous pulsing of mouse spleen DCs with a relevant vaccine protein, such as anti-DEC-205 HIV gag p24. We successfully demonstrated that VDRFYKTLRAQASQ (D6) and DRFYKTLRAQASQ (D6.1) peptides are two naturally processed epitopes from the entire HIV gag p24 protein (Figure 4 and 5), when delivered to DCs through the DEC-205 receptor [3], [12], [13], [14]. We found that DCs display relatively small amounts of naturally processed VDRFYKTLRAQASQ (D6) and DRFYKTLRAQASQ (D6.1) MHC II peptides derived from anti-DEC-205 HIV gag p24 protein (Figure 5). Nonetheless, the identified MHC II-associated HIV gag p24 peptides elicited strong dose-dependent responses from antigen specific CD4^+^ T cells, which have acquired high avidity for these MHC II-bound peptides as a result of the immunization with anti-DEC 205-HIV gag p24 protein (Figure 6 and Figure S8) [33]. These observations are consistent with previously published work suggesting that T cell activation correlates inversely with the number of MHC complexes of cognate peptide per DC and that very few MHC-peptide complexes are necessary for the induction of full-fledged T cell activation [34], [35], [36].

As demonstrated above, a major advantage of the described strategy is the ability to identify distinct MHC-bound peptides from antigenic, vaccine proteins. In the experiments presented in this manuscript we focused our efforts on identifying the two immunodominant (D6) and DRFYKTLRAQASQ (D6.1) peptides derived from the HIV gag p24 protein. Future experiments will examine whether additional peptides derived from the exogenously added DEC-205 HIV gag p24 protein are presented on mouse spleen DCs following the delivery of the HIV gag p24 protein through the DEC-205 receptor.

When VDRFYKTLRAQASQ peptide was added exogenously to DCs *in vitro* its presentation required intracellular processing (Figure 3C and 6B), suggesting an active role of DCs in generating MHC II-bound peptides from an exogenous peptide source [23], [37] and not a direct binding of peptides to HLA molecules at the cell surface [22], [38]. Three out of the four HIV gag p24 mimetopes, added exogenously, were not detected by LC-MS/MS (Figure S1) and did not elicit proliferation of CD4^+^ T cells measured by a CFSE dilution assay (Figure S6).

Application of MS analysis for identification of antigen-derived peptides presented on HLA molecules is becoming a major focus of vaccine development [32]. Naturally processed and presented peptides can be used as vaccine candidates - either as multi epitope-based vaccine [11] or DC-based vaccine candidates [12], [13], [39] – which offer the advantages of high immunogenicity and valuable efficacy [40]. Recently, Ranasinghe et al. reported that an extended HIV gag D6 peptide sequence, YVDRFYKTLRAQASQEV (amino acid 296–313), was frequently recognized among HIV infected subjects (40% of responders) and associated with spontaneous control of disease progression, independently of the patient HLA haplotype [41]. Furthermore, it has been demonstrated that sequence conservation in this region results in a high degree of cross-clade CD4^+^ T cell recognition [42] and elicits high avidity CD4^+^ T cells [33].

In conclusion, the present strategy promises to assist vaccine research by facilitating the identification and selection of vaccine-derived epitopes. The direct characterization of T cell epitopes opens up a powerful new approach to assess the efficacy of different vaccination strategies, formulations, antigenic inserts and adjuvants as well as new robust method for clinical trial monitoring. This advance will allow us to correlate the peptide repertoire presented on DCs with the repertoire of T cell responses elicited in vaccine recipients [12]. In turn, this understanding should lead to an accelerated rational design of clinical HIV vaccine.

## Materials and Methods

Detailed materials and methods are provided in Materials and Methods S1.

### Ethics Statement

All procedures were approved by the Institutional Animal Care and Use Committee (IACUC) at The Rockefeller University, New York (Approval ID numbers 11478 and 11460). Balb/c×C57Bl/6 (I-A^b,d^/Ed) F1 mice were used in this study. Animals were maintained under specific pathogen-free conditions and used at 6–8 wk of age in accordance with the IACUC guidelines.

### Cell Preparation

CD11c^+^ DCs were obtained from Flt3L-treated Balb/c×C57Bl/6 (I-A^b,d^/E^d^) F1 mice [7], [16]. Total 20–30×10^6^ purified DC per well were pulsed with 1 µM of synthetic 15-mer peptides or 1 µg/ml (5 pM) of anti-DEC-205 HIV gag p24 protein for 5–6 h. Twenty five µg/mL polyinosinic:polycytidylic acid (poly IC) was also added as a maturation stimulus. In some experiments, 20 mM of NH_4_Cl or 100 µM CQ was used to preincubate DCs. Treated and untreated DCs were loaded with peptides.

### Affinity Purification of MHC II-peptide Complexess and Peptides Purification

MHC II molecules were purified by immunoaffinity from cell lysate [7]. The MHC II peptide complexes were eluted with 10% acetic acid [7]. Purified MHC II peptides were dried and reconstituted in 20 µL 0.1% TFA/water. Half of the peptide mixture (3.5–4×10^8^ cell equivalents) was injected for LC-MS analysis.

### LC-MS Analysis

We used a Dionex U3000 capillary/nano-HPLC system (ThermoFisher Scientific, San Jose, CA) interfaced with the Thermo Fisher LTQ-Orbitrap mass spectrometer, operated in a data-dependent acquisition mode cycling through a single MS full-scan (620–1200 m/z, 30,000 resolution) followed by 6 MS/MS scans in the ion trap [7].

### EpiSifter

EpiSifter was implemented using Perl (www.perl.org) and R (www.r-project.org). First, sequences of the proteins of interest are cut to generate all possible MHC II peptides, and corresponding m/z’s are calculated for charge states +1 to +4, considering oxidized and unoxidized methionine. Second, the mass chromatograms for the possible peptides, modifications, charge states, and isotopic peaks, and interfering peaks are extracted from the data within the mass accuracy of the mass spectrometer (10 ppm). Third, the mass chromatograms are plotted together with the expected intensity ratio’s using R.

### Quantitation of MHC II Peptides

MHC II peptide mixture (10 µL) in 0.1% TFA/water was mixed with 10 µL of a solution containing known amounts (1 ng) of the two synthetic isotope labeled peptides dissolved in 50% acetonitrile/0.1% TFA/water. The entire peptide mixture was subjected to LC-MS analysis [7].

### T Cell Cultures

Antigen-specific bulk splenocytes were isolated from F1 mice primed intraperitoneally with 5 µg anti-DEC-205 HIV gag p24 protein and 50 µg of polyinosinic-polycytidylic acid stabilized with polylysine and carboxymethylcellulose (poly ICLC), as adjuvant, and boosted under the same condition 4–6 weeks later [21]. Spleens from immunized mice (2–4 mice) were collected 2 weeks later after boost. CFSE-labeled bulk splenocytes were cultured with either 0.05 µg/ml of HIV gag p24 peptide mix, HIV gag D6 and D6.1 peptides, HIV gag p17 or ApoE peptide (KELEEQLGPVAEETR) [7]. For DC:T cell cocultures, 2×10^6^ splenic CD11c^+^ DCs were pulsed with poly IC and different HIV gag p24 peptide concentrations. NH_4_Cl-, CQ-treated or untreated DCs were loaded with peptides, washed and added to CFSE labeled HIV gag p24 primed T cells. After 4 days of culture, cell proliferation and cytokine production were determined by intracellular cytokine staining and FACS analysis.

## Supporting Information

Figure S1 **Query of DC MHC II- bound HIV gag p24 peptides after DCs were pulsed** ***in vitro*** **with a peptide mixture.** Panels A, B and C show the full scan generated by EpiSifter for the 3 HIV gag mimetopes (Table 1). Note that no signals with correct m/z and isotope distribution ratios were detected (compare to Figure 2).
(PPTX)

Figure S2 **Identification of MHC II-bound HIV gag p24 peptides.** Comparison of MS/MS spectra of eluted HIV gag p24 peptides VDRFYKTLRAEQASQ (m/z 906.4745, z = 2, Figure 3A) (A) and DRFYKTLRAEQASQ (m/z 856.9398, z = 2, Figure 3B) (B) with MS/MS spectra of the corresponding synthetic isotopically labeled peptides (m/z 909.9822, z = 2, Table S3) (C) and (m/z 860.4479, z = 2, Table S3) (D). The isotopically labeled amino acid is labeled with a (*). The corresponding y and b series are marked.
(PPTX)

Figure S3 **NH_4_Cl inhibits MHC II- binding of HIV gag p24 peptides VDRFYKTLRAQASQ and DRFYKTLRAQASQ.** (A, B) The same experiment as in Figure 3A and 3B was performed in the presence of 20 mM of NH_4_Cl. No peptide ions (m/z = 906.4736 and m/z = 856.9394) corresponding to the peptides above could be detected.
(PPTX)

Figure S4 **Quantitation of MHC II-associated VDRFYKTLRAEQASQ and DRFYKTLRAEQASQ HIV gag p24 peptides.** Heavy isotopes peaks are indicated with a (*). MS profile of VDRFYKTLRAEQASQ (A) and DRFYKTLRAEQASQ (B) isotope peptide pair identified as MHC II-bound peptides. The detected m/z values are indicated in bold letters.
(PPTX)

Figure S5 **Quantitation analysis of a selected endogenous MHC II peptide.** Heavy isotopes peaks are indicated with a (*). MS profile of the ApoE isotope peptide pair (KELEEQL*GPVAEETR, mass shift 1727.88+7 Da) identified in the MHC II peptide mixture eluted from the experiment shown in Figure 5. The detected m/z values are indicated in bold letters.
(PPTX)

Figure S6 **Immuno-reactivity of HIV gag p24 mimetopes measured by FACS analysis.** CFSE-labeled bulk splenocytes from HIV gag p24 immunized mice were stimulated with negative control HIV gag p17 peptide mix, positive control HIV gag p24 peptide mix and with the four HIV gag p24 mimetopes listed in Table 1.
(PPTX)

Figure S7 **Presentation of HIV gag p24 VDRFYKTLRAEQASQ peptide in DC:T cell cocultures.** Untreated (medium) and NH_4_Cl-treated DCs, prepared from the experiment described in Figure 3A and 3C, were loaded with graded doses of VDRFYKTLRAEQASQ peptide, washed with PBS and added to CFSE-labeled purified T cells. Values represent mean ± SD of the percentage of CD4^+^ IFNγ^+^ T cells pooled from two independent experiments.
(PPTX)

Figure S8 **Functional avidity of MHC II–bound HIV gag p24 peptides in DC:T cell cocultures.** For each peptide concentration (−logM), the frequencies of CD4^+^ IFNγ^+^ T cells measured in (Figure 6B) were expressed as percentage of the maximum response obtained at saturation (1×10^−6^ M). The indicated EC_50_ represents the peptide concentration of VDRFYKTLRAEQASQ and DRFYKTLRAEQASQ peptides, respectively, required to attain 50% of maximal IFNγ production by HIV gag p24 specific CD4^+^ T cells.
(PPTX)

Table S1 **List of all theoretically predicted peptides, m/z ratios and relative intensities for the isotope distributions, calculated by EpiSifter from the HIV gag p24 mimetope sequences shown in** **Table 1** **.**
(XLS)

Table S2 **List of identified DC MHC II-bound peptides.** Peptide sequences were analyzed by tandem MS and identified by searching the IPI database for mouse protein sequences plus the HIV gag p24 protein sequence using the X! Tandem algorithm (see Materials and Methods), allowing for enzymatic cleavage between any pair of amino acids. Only peptides with mass accuracy better than 20 ppm and 0.4 Da for precursors and fragments, respectively, were included.
(XLS)

Table S3 **List of isotope labeled peptides synthesized for quantitation analysis and their MS parameters.** ^(a)^Mass corresponds to the monoisotopic form of the selected peptide. ^(b)^Predicted mass shift due to the incorporation of the isotopically labeled amino acid.
(DOCX)

Table S4 **List of all theoretically predicted peptides, m/z ratios and relative intensities for the isotope distributions, calculated by EpiSifter from the HIV gag p24 protein sequence (aa 133–363 derived from HIV isolate BH10).**
(XLSX)

Materials and Methods S1 **Detailed materials and methods.**
(DOCX)
