# Supplementary figures and images for: Strategy for Identifying Dendritic Cell-Processed CD4+ T Cell Epitopes from the HIV Gag p24 Protein
Source: PLoS One. 2012 Jul 30;7(7):e41897. doi: 10.1371/journal.pone.0041897 (PMC3408443; doi:10.1371/journal.pone.0041897)

## Slide 1
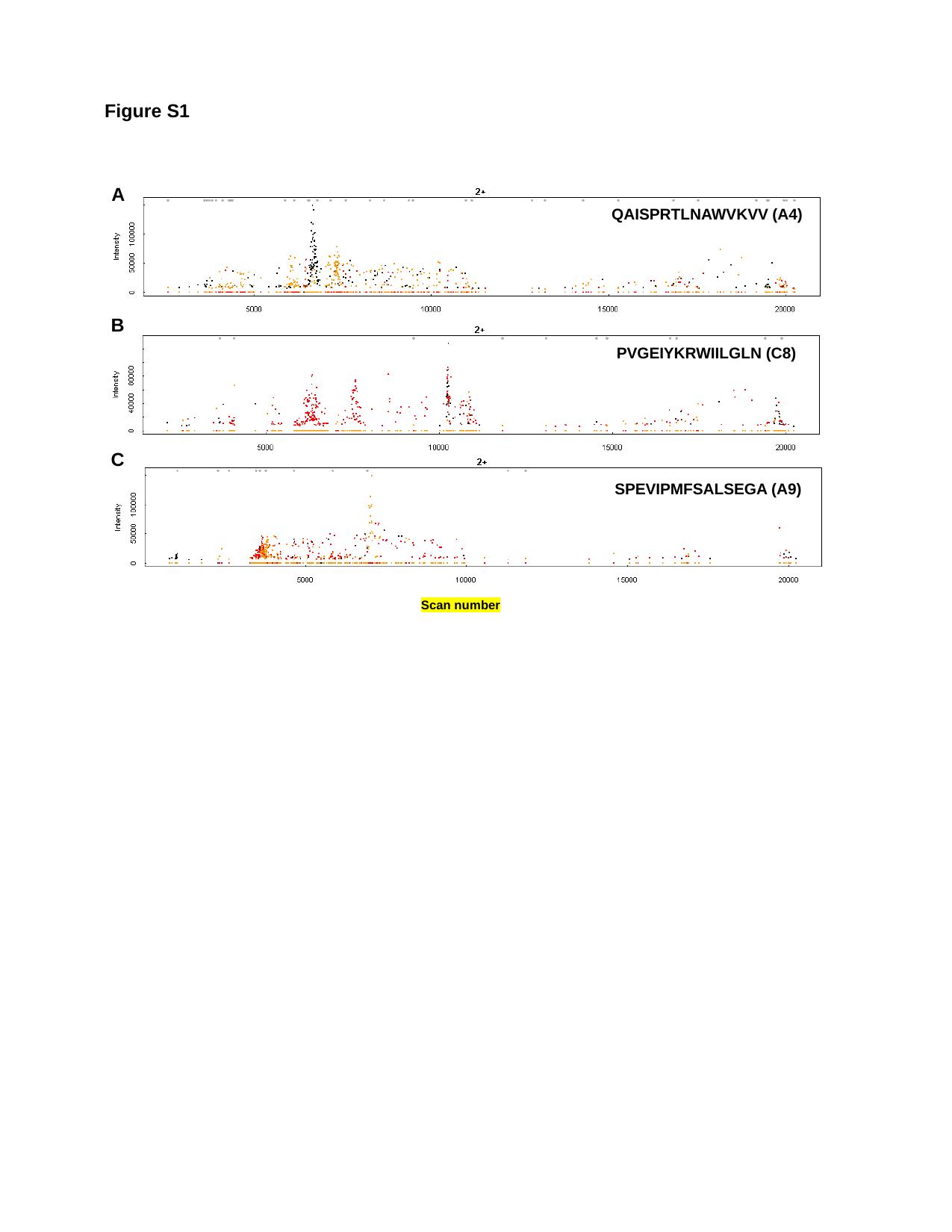

Figure S1
A
QAISPRTLNAWVKVV (A4)
B
PVGEIYKRWIILGLN (C8)
C
SPEVIPMFSALSEGA (A9)
Scan number

Supplement: Figure S1 — Query of DC MHC II- bound HIV gag p24 peptides after DCs were pulsed in vitro with a peptide mixture. Panels A, B and C show the full scan generated by EpiSifter for the 3 HIV gag mimetopes (Table 1). Note that no signals with correct m/z and isotope distribution ratios were detected (compare to Figure 2). (PPTX) [file pone.0041897.s001.pptx]

## Slide 1
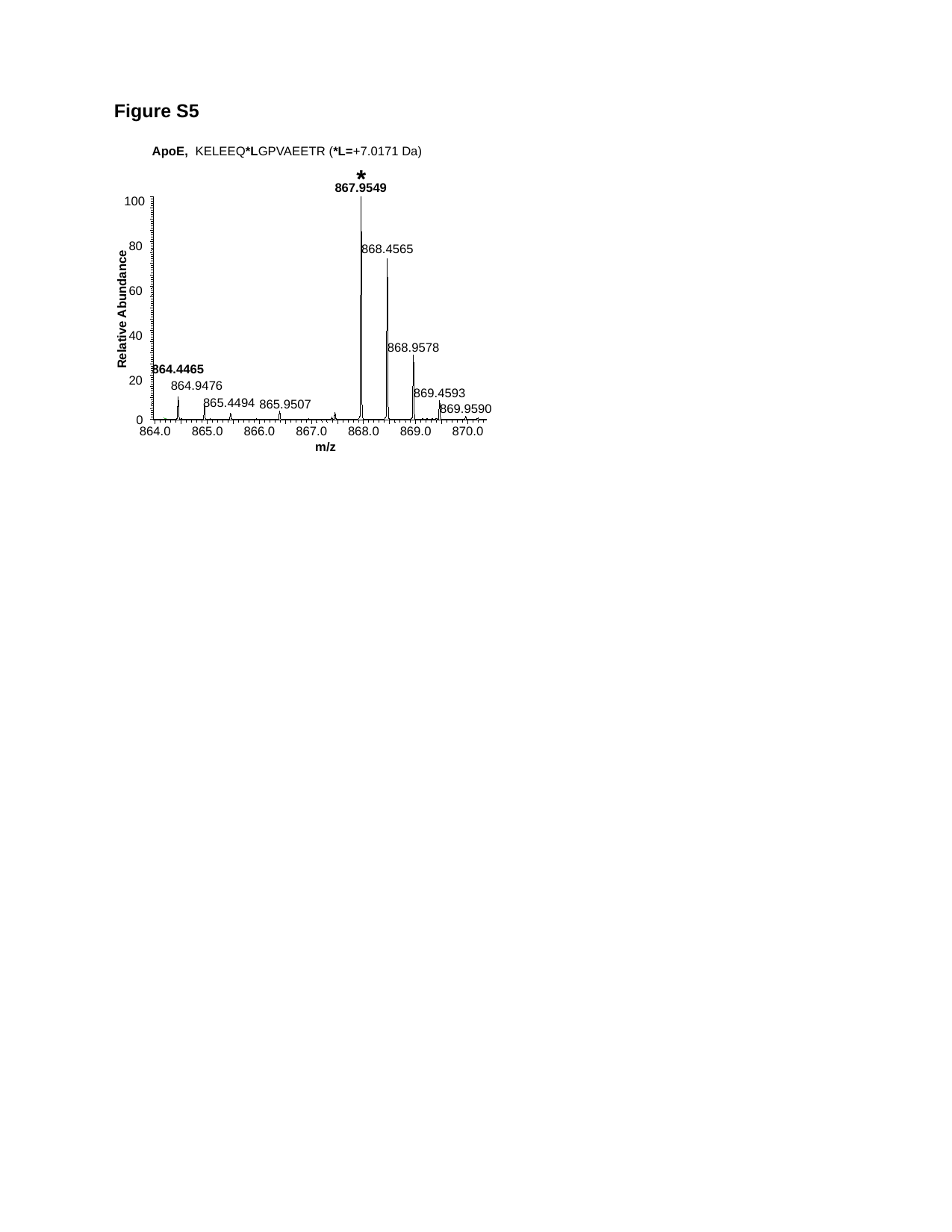

Figure S5
ApoE, KELEEQ*LGPVAEETR (*L=+7.0171 Da)
*
867.9549
100
80
868.4565
Relative Abundance
60
40
868.9578
864.4465
20
864.9476
869.4593
865.4494
865.9507
869.9590
0
864.0
865.0
866.0
867.0
868.0
869.0
870.0
m/z

Supplement: Figure S5 — Quantitation analysis of a selected endogenous MHC II peptide. Heavy isotopes peaks are indicated with a (*). MS profile of the ApoE isotope peptide pair (KELEEQL*GPVAEETR, mass shift 1727.88+7 Da) identified in the MHC II peptide mixture eluted from the experiment shown in Figure 5. The detected m/z values are indicated in bold letters. (PPTX) [file pone.0041897.s005.pptx]

## Slide 1
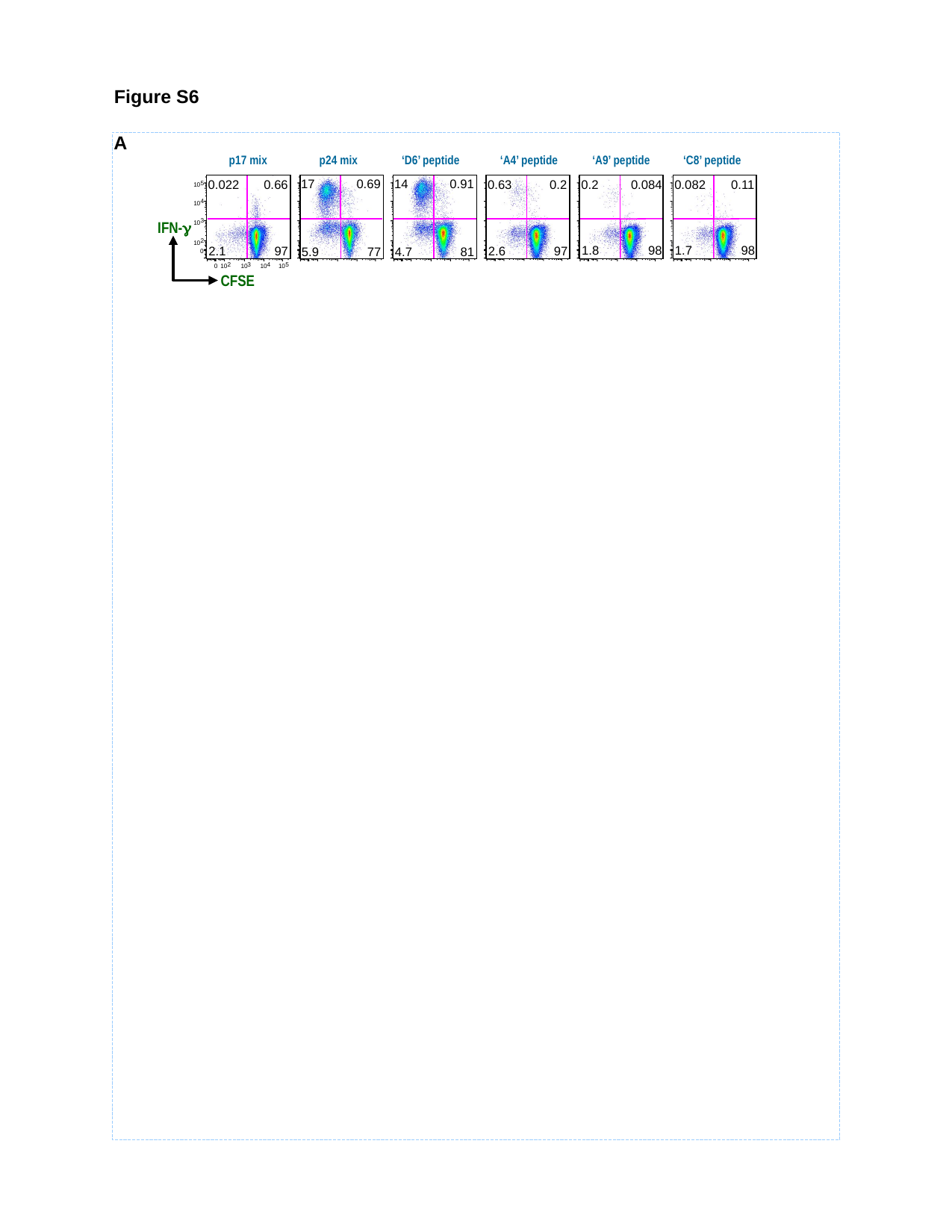

Figure S6
A
p17 mix
p24 mix
‘D6’ peptide
‘A4’ peptide
‘A9’ peptide
‘C8’ peptide
17
0.69
14
0.91
0.022
0.66
0.63
0.2
0.082
0.11
0.2
0.084
5
10
4
10
3
10
2
10
0
IFN-γ
CFSE
1.7
98
1.8
98
2.1
97
2.6
97
4.7
81
5.9
77
2
3
4
5
0
10
10
10
10

Supplement: Figure S6 — Immuno-reactivity of HIV gag p24 mimetopes measured by FACS analysis. CFSE-labeled bulk splenocytes from HIV gag p24 immunized mice were stimulated with negative control HIV gag p17 peptide mix, positive control HIV gag p24 peptide mix and with the four HIV gag p24 mimetopes listed in Table 1. (PPTX) [file pone.0041897.s006.pptx]

## Slide 1
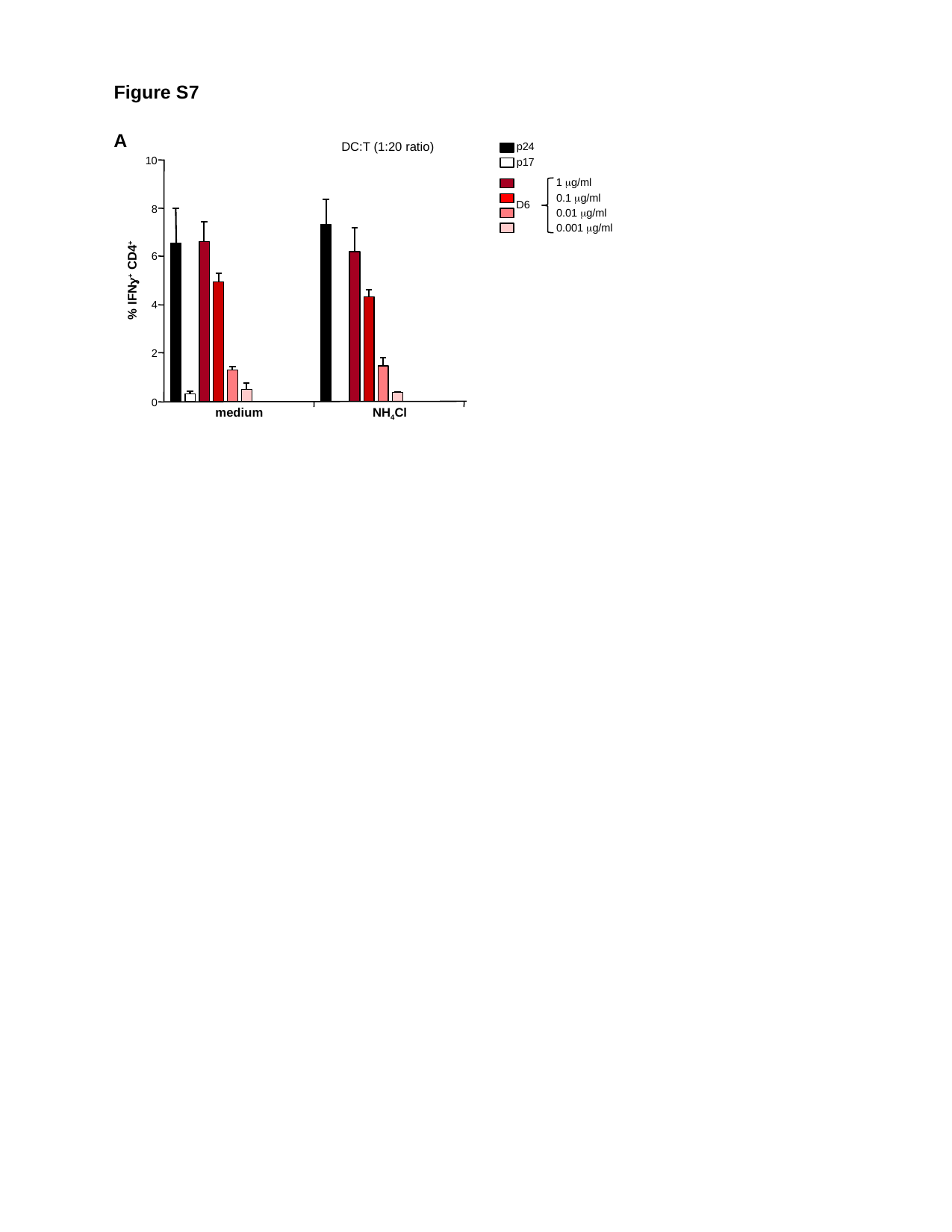

Figure S7
A
DC:T (1:20 ratio)
p24
p17
1 mg/ml
0.1 mg/ml
D6
0.01 mg/ml
0.001 mg/ml
10
8
6
% IFNg+ CD4+
4
2
0
medium
NH4Cl

Supplement: Figure S7 — Presentation of HIV gag p24 VDRFYKTLRAEQASQ peptide in DC:T cell cocultures. Untreated (medium) and NH4Cl-treated DCs, prepared from the experiment described in Figure 3A and 3C, were loaded with graded doses of VDRFYKTLRAEQASQ peptide, washed with PBS and added to CFSE-labeled purified T cells. Values represent mean ± SD of the percentage of CD4+ IFNγ+ T cells pooled from two independent experiments. (PPTX) [file pone.0041897.s007.pptx]
