# Supplementary material for: Strategy for Identifying Dendritic Cell-Processed CD4+ T Cell Epitopes from the HIV Gag p24 Protein
Source: PLoS One. 2012 Jul 30;7(7):e41897. doi: 10.1371/journal.pone.0041897 (PMC3408443; doi:10.1371/journal.pone.0041897)
Supplement: Figure S2 — Identification of MHC II-bound HIV gag p24 peptides. Comparison of MS/MS spectra of eluted HIV gag p24 peptides VDRFYKTLRAEQASQ (m/z 906.4745, z = 2, Figure 3A) (A) and DRFYKTLRAEQASQ (m/z 856.9398, z = 2, Figure 3B) (B) with MS/MS spectra of the corresponding synthetic isotopically labeled peptides (m/z 909.9822, z = 2, Table S3) (C) and (m/z 860.4479, z = 2, Table S3) (D). The isotopically labeled amino acid is labeled with a (*). The corresponding y and b series are marked. (PPTX) [file pone.0041897.s002.pptx]

## Slide 1
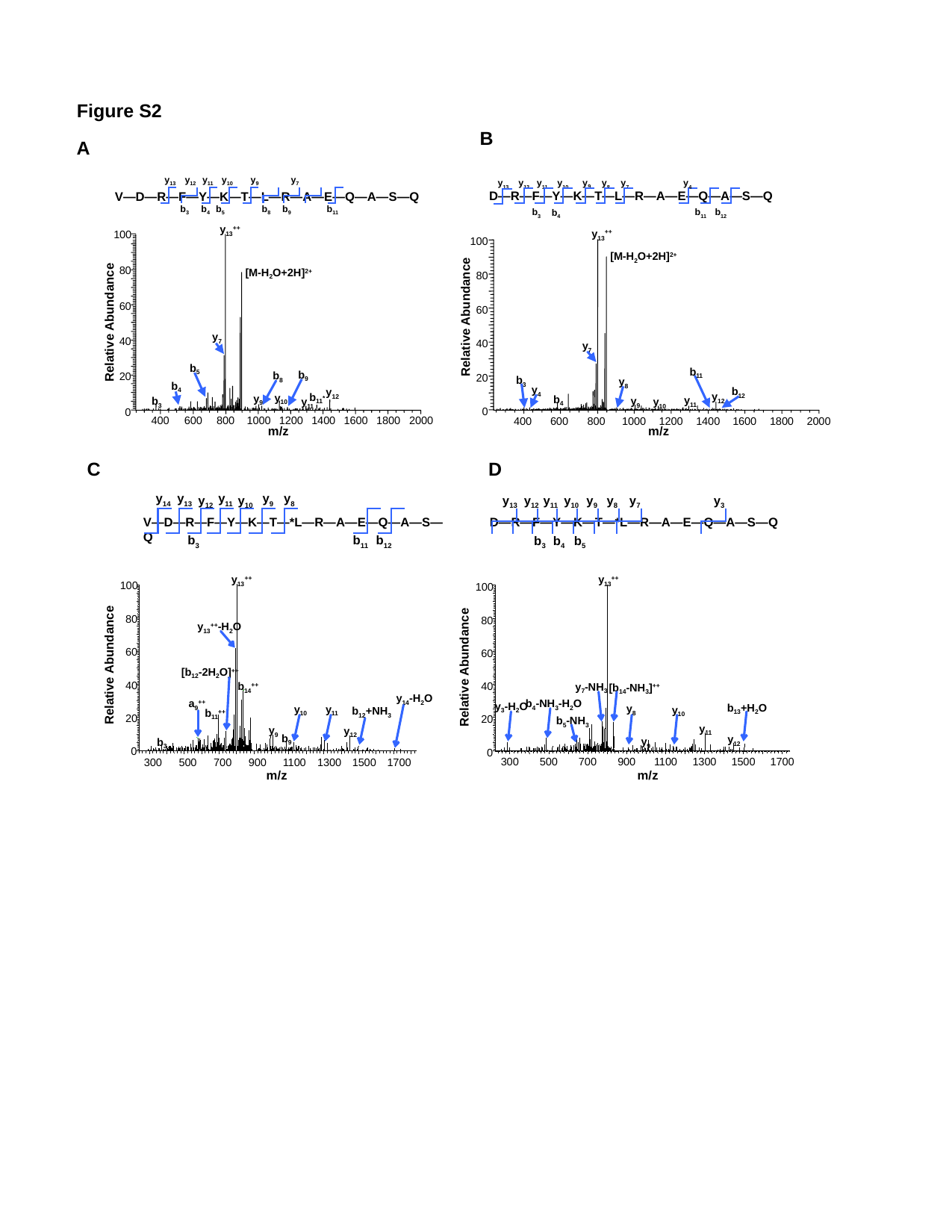

Figure S2
B
A
y12
y13
y11
y10
y9
y7
V—D—R—F—Y—K—T—L—R—A—E—Q—A—S—Q
b3
b4
b5
b8
b9
b11
y9
y11
y10
y8
y7
y4
y12
y13
D—R—F—Y—K—T—L—R—A—E—Q—A—S—Q
b3
b11
b12
b4
y13++
100
[M-H2O+2H]2+
80
60
Relative Abundance
40
y7
b11
20
b3
y8
y4
b12
y12
b4
y11
y9
y10
0
400
600
800
1000
1200
1400
1600
1800
2000
m/z
y13++
[M-H2O+2H]2+
y7
b5
b9
b8
b4
y12
b11*
y10
y9
b3
y11
100
80
60
Relative Abundance
40
20
0
400
600
800
1000
1200
1400
1600
1800
2000
m/z
C
D
y14
y13
y11
y9
y8
y12
y10
V—D—R—F—Y—K—T—*L—R—A—E—Q—A—S—Q
b3
b11
b12
y13
y12
y11
y10
y9
y8
y7
y3
 D—R—F—Y—K—T—*L—R—A—E—Q—A—S—Q
b3
b4
b5
y13++
100
80
60
Relative Abundance
y7-NH3
[b14-NH3]++
40
b4-NH3-H2O
y3-H2O
b13+H2O
y8
y10
b5-NH3
20
y11
y12
y9
0
300
500
700
900
1100
1300
1500
1700
m/z
y13++
100
80
y13++-H2O
60
Relative Abundance
[b12-2H2O]++
b14++
40
y14-H2O
a9++
y10
y11
b12+NH3
b11++
20
y9
y12
b9
b3
0
300
500
700
900
1100
1300
1500
1700
m/z
