# Supplementary material for: Strategy for Identifying Dendritic Cell-Processed CD4+ T Cell Epitopes from the HIV Gag p24 Protein
Source: PLoS One. 2012 Jul 30;7(7):e41897. doi: 10.1371/journal.pone.0041897 (PMC3408443; doi:10.1371/journal.pone.0041897)
Supplement: Figure S3 — NH4Cl inhibits MHC II- binding of HIV gag p24 peptides VDRFYKTLRAQASQ and DRFYKTLRAQASQ. (A, B) The same experiment as in Figure 3A and 3B was performed in the presence of 20 mM of NH4Cl. No peptide ions (m/z = 906.4736 and m/z = 856.9394) corresponding to the peptides above could be detected. (PPTX) [file pone.0041897.s003.pptx]

## Slide 1
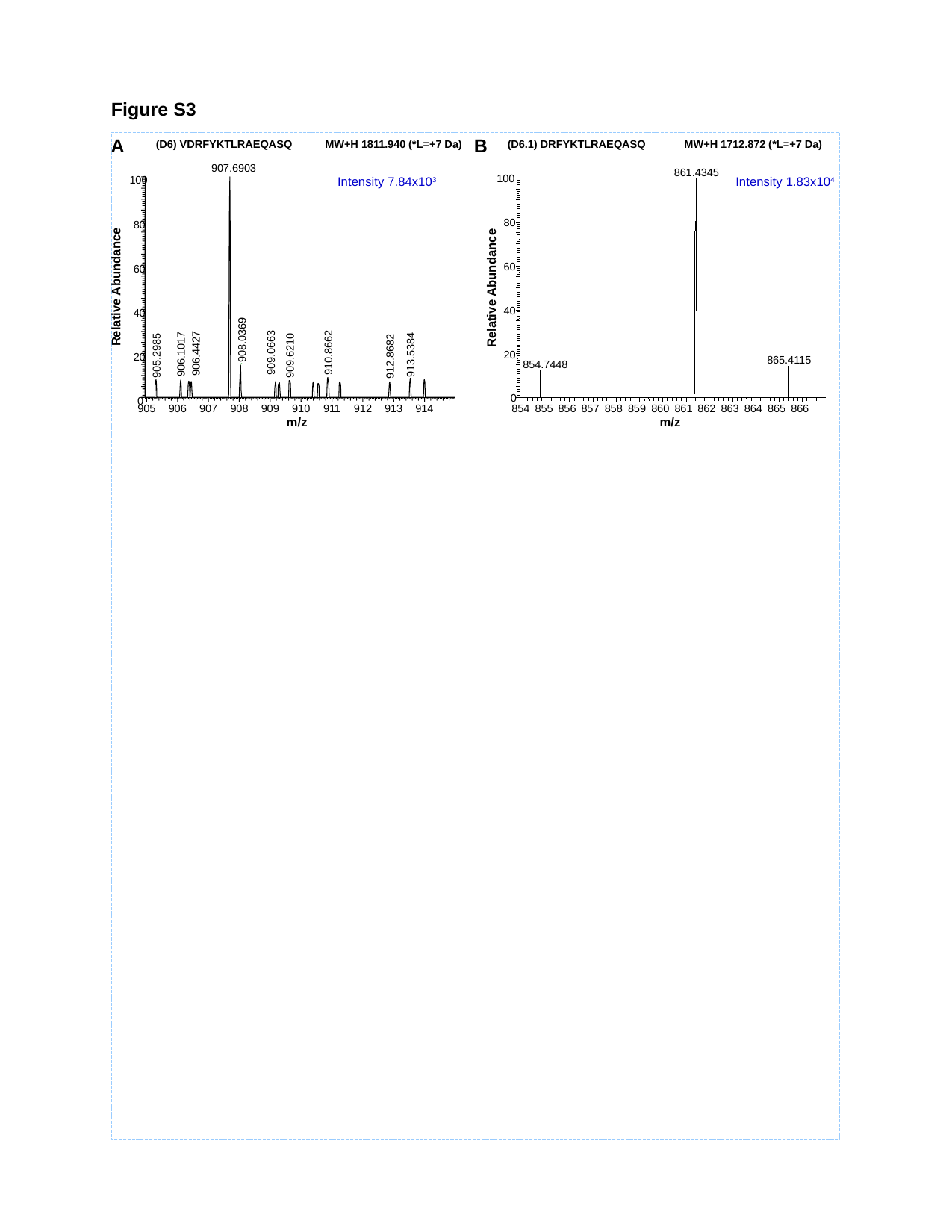

Figure S3
A
B
(D6) VDRFYKTLRAEQASQ MW+H 1811.940 (*L=+7 Da)
(D6.1) DRFYKTLRAEQASQ MW+H 1712.872 (*L=+7 Da)
907.6903
100
80
60
Relative Abundance
40
908.0369
909.0663
910.8662
906.4427
906.1017
913.5384
905.2985
909.6210
912.8682
20
0
905
906
907
908
909
910
911
912
913
914
m/z
861.4345
100
80
60
Relative Abundance
40
20
865.4115
854.7448
0
854
855
856
857
858
859
860
861
862
863
864
865
866
m/z
Intensity 7.84x10^3^
Intensity 1.83x10^4^
