# Supplementary material for: Strategy for Identifying Dendritic Cell-Processed CD4+ T Cell Epitopes from the HIV Gag p24 Protein
Source: PLoS One. 2012 Jul 30;7(7):e41897. doi: 10.1371/journal.pone.0041897 (PMC3408443; doi:10.1371/journal.pone.0041897)
Supplement: Figure S4 — Quantitation of MHC II-associated VDRFYKTLRAEQASQ and DRFYKTLRAEQASQ HIV gag p24 peptides. Heavy isotopes peaks are indicated with a (*). MS profile of VDRFYKTLRAEQASQ (A) and DRFYKTLRAEQASQ (B) isotope peptide pair identified as MHC II-bound peptides. The detected m/z values are indicated in bold letters. (PPTX) [file pone.0041897.s004.pptx]

## Slide 1
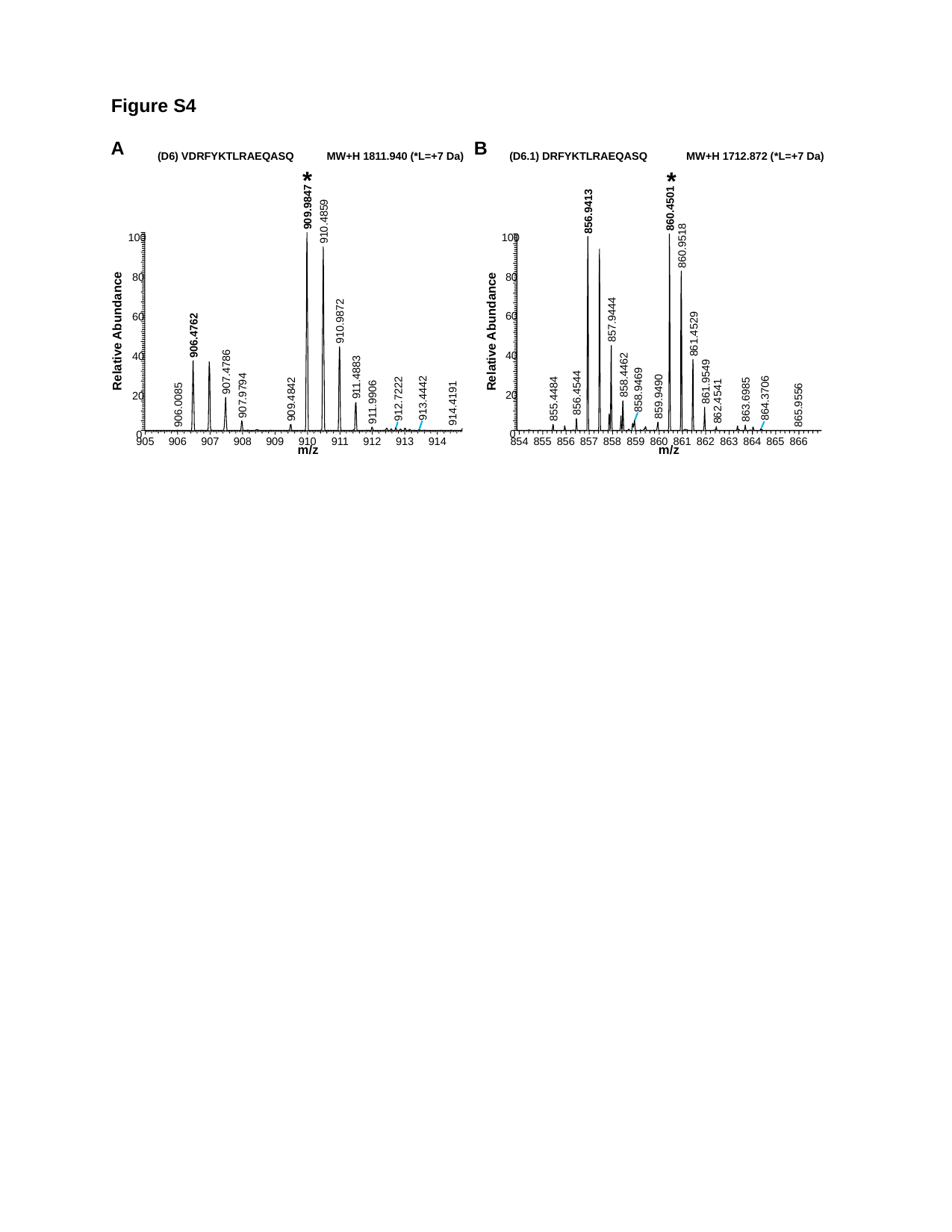

Figure S4
A
B
(D6) VDRFYKTLRAEQASQ MW+H 1811.940 (*L=+7 Da)
(D6.1) DRFYKTLRAEQASQ MW+H 1712.872 (*L=+7 Da)
*
*
909.9847
910.4859
100
80
60
910.9872
Relative Abundance
906.4762
40
907.4786
911.4883
20
907.9794
913.4442
912.7222
909.4842
911.9906
914.4191
906.0085
0
905
906
907
908
909
910
911
912
913
914
m/z
860.4501
856.9413
100
860.9518
80
60
857.9444
Relative Abundance
861.4529
40
858.4462
861.9549
858.9469
856.4544
20
859.9490
864.3706
855.4484
863.6985
862.4541
865.9556
0
854
855
856
857
858
859
860
861
862
863
864
865
866
m/z
