# Supplementary material for: Strategy for Identifying Dendritic Cell-Processed CD4+ T Cell Epitopes from the HIV Gag p24 Protein
Source: PLoS One. 2012 Jul 30;7(7):e41897. doi: 10.1371/journal.pone.0041897 (PMC3408443; doi:10.1371/journal.pone.0041897)
Supplement: Figure S8 — Functional avidity of MHC II–bound HIV gag p24 peptides in DC:T cell cocultures. For each peptide concentration (−logM), the frequencies of CD4+ IFNγ+ T cells measured in (Figure 6B) were expressed as percentage of the maximum response obtained at saturation (1×10^−6^ M). The indicated EC50 represents the peptide concentration of VDRFYKTLRAEQASQ and DRFYKTLRAEQASQ peptides, respectively, required to attain 50% of maximal IFNγ production by HIV gag p24 specific CD4+ T cells. (PPTX) [file pone.0041897.s008.pptx]

## Slide 1
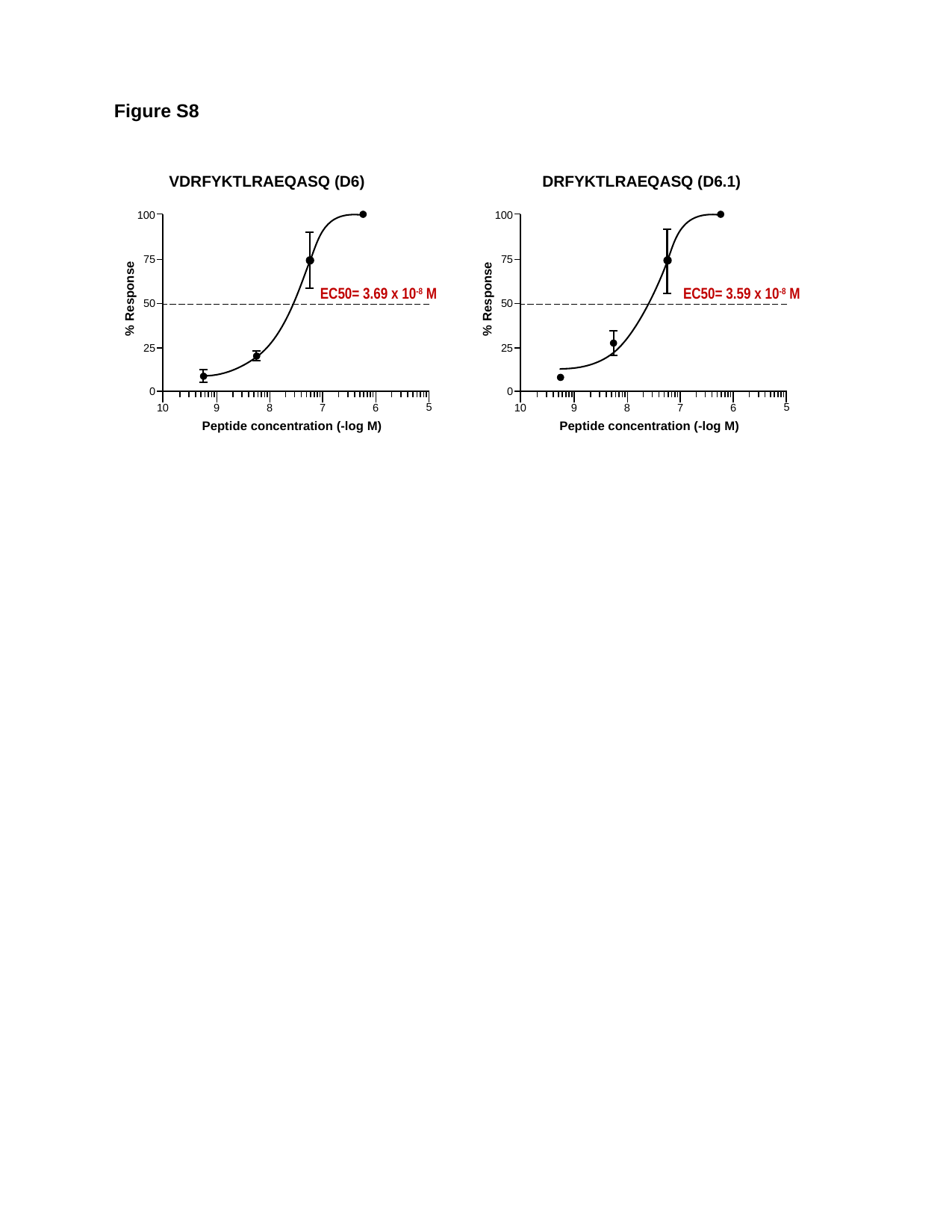

Figure S8
VDRFYKTLRAEQASQ (D6)
DRFYKTLRAEQASQ (D6.1)
100
75
% Response
50
25
0
5
10
9
8
7
6
Peptide concentration (-log M)
100
75
% Response
50
25
0
5
10
9
8
7
6
Peptide concentration (-log M)
EC50= 3.69 x 10^-8^ M
EC50= 3.59 x 10^-8^ M
