# Supplementary material for: Strategy for Identifying Dendritic Cell-Processed CD4+ T Cell Epitopes from the HIV Gag p24 Protein
Source: PLoS One. 2012 Jul 30;7(7):e41897. doi: 10.1371/journal.pone.0041897 (PMC3408443; doi:10.1371/journal.pone.0041897)
Supplement: Table S3 — List of isotope labeled peptides synthesized for quantitation analysis and their MS parameters. (a)Mass corresponds to the monoisotopic form of the selected peptide. (b)Predicted mass shift due to the incorporation of the isotopically labeled amino acid. (DOCX) [file pone.0041897.s011.docx]

**Table S3. List of isotopically labeled peptides synthesized for quantitation analysis and their MS parameters**

| **HIV gag p24 peptide** | | | **Isotopically labeled peptide sequence** | | |
| --- | --- | --- | --- | --- | --- |
|  | **MW (Da)** | **m/z (z=2)** |  | **Mass shift (+ 7.0171 Da)** | **m/z (z=2)** |
| **HIV gag D6 (aa 297-311)** | 1811.9399^a^ | 906.4736 | VDRFYKT***L**RAEQASQ | 1818.9570^b^ | 909.9822 |
| **HIV gag D6.1 (aa 298-311)** | 1712.8715^a^ | 856.9394 | DRFYKT***L**RAEQASQ | 1719.8886^b^ | 860.4479 |
