# Supplementary material for: Strategy for Identifying Dendritic Cell-Processed CD4+ T Cell Epitopes from the HIV Gag p24 Protein
Source: PLoS One. 2012 Jul 30;7(7):e41897. doi: 10.1371/journal.pone.0041897 (PMC3408443; doi:10.1371/journal.pone.0041897)
Supplement: Materials and Methods S1 — Detailed materials and methods. (DOCX) [file pone.0041897.s013.docx]

## Supplemental Materials and Methods

## Mice

Balb/c x C57Bl/6 (I-A^b,d^/E^d^) F1 mice from Harlan Animal Research Laboratory (3565 Paysphere Circle, Chicago, IL 60674 USA) were maintained under specific pathogen-free conditions and used at 6-8 wk of age in accordance with Rockefeller University Animal Care and Use Committee guidelines.

## Cell lines, Antibodies, Reagents

Melanoma cells expressing Fms-like tyrosine kinase 3 ligand (Flt3L) were established via retroviral gene transfer([1](#_ENREF_1)) and generously provided by L. Santambrogio (Albert Einstein College of Medicine, New York, NY). B16 Flt3L melanoma cells were cultured with DMEM containing 10% FBS and 5 x 10^6^ were injected s.c into the abdomen region of mice. After 15-20 days, all major splenic DC subsets had expanded >10 fold in agreement with previous reports([2](#_ENREF_2), [3](#_ENREF_3)). The anti-MHC class II (N22) hydridoma cells([4](#_ENREF_4)) were maintained in DMEM medium with 2 mM L-glutamine, 5% heat-inactivated FBS and 1% penicillin-streptomycin. The N22 monoclonal antibody was affinity-purified from culture supernatants using Protein G Sepharose (Amersham Biosciences). Poly IC (polyinosinic:polycytidylic acid) was from Thermo Scientific (Waltham, MA, USA). Ammonium chloride (NH_4_Cl) and chloroquine (CQ) were purchased from Sigma (St. Louis, Mo).

**Cell preparation**

Flt3L-treated spleens were removed, cut in small fragments, and digested into single cell-suspensions with 400 U/ml collagenase D (Roche Applied Science) for 25 min at 37^0^C. After inhibition of collagenase with 10 mM EDTA, cells were resuspended in PBS in 2 mM EDTA and 2% FCS. CD11c^+^ DC were enriched by positive selection using anti-CD11c magnetic beads and MACS columns (Miltenyi Biotec). From a pool of 12-17 mice we could typically obtain from 5x10^8^ to 7x10^8^ DCs. For MHC II peptide analysis, a total of 20-30 x10^6^ purified DC per well were pulsed with 1 µM of synthetic 15-mer peptides or 1 μg/ml (5 pM) of anti-DEC-205 HIV gag p24 protein for 5-6 h in 6-well culture plates in a final volume of 3 mL RPMI 1640 containing 5% FBS and the supernatant (3% vol/vol) from J558L cells transduced with murine GM-CSF (complete medium). Twenty five μg/mL poly IC was also added to the cultures as *in vitro* maturation stimulus. Then DCs were washed three times with PBS and readied for cell lysis.

For immunoassays, antigen-primed bulk splenocytes or T cells were isolated from F1 mice primed and boosted with anti DEC-HIV gag p24 and poly IC([5](#_ENREF_5)). An enriched preparation of HIV gag p24 specific T cells was obtained by excluding MHC class II+ cells, B cells and macrophages using respectively TIB120/M5/114, B220 TIB 146/ RA3-3A1/6.1 and F4/80 rat mAbs([3](#_ENREF_3)). For preparation of DCs as stimulators in DC:T cells coculture, 2x10^6^ of DCs were resuspended in 1ml of complete medium, pulsed with peptides and matured with 25 μg/ml of poly IC for 4-5h as above. After, extensive wash T cells were added to 96-well U bottom plate at a DC:T cell ratio of 1:20.

In some experiments, 20 mM of NH_4_Cl or 100 µM chloroquine were added as lysosomotropic drugs to study peptide processing. In this case, a fraction of DCs were preincubated in the presence of these inhibitors for 30 min at 37^0^C. Treated and untreated DCs were loaded with peptides for additional 4 h at 37^0^C, washed with PBS and readied for peptide analysis or T cell assays.

## Affinity purification of MHC II molecules

MHC II molecules were purified by immunoaffinity from the cells after lysis with 1% CHAPS (Anatrace), 0.1 mM iodoacetamide, 5 mM EDTA, 1:100 Protease Inhibitors Cocktail, 1 mM PMSF (Sigma) in 20 mM Tris-HCl pH 8 at 4 °C for 45 min on a rotator. The lysate was cleared by 30 min centrifugation at 15000 RPM. MHC class II molecules from cleared lysate were immunoaffinity purified with 12- 15 mg of purified antibody N22 bound to CNBr-activated Sepharose (GE Healthcare) at a ratio of 40 mg Sepharose per mg of antibody following manufacturer’ s protocol. The affinity column was washed first with 3 column volumes of lysis buffer, followed by 6 column volumes of 250 mM NaCl, 50 mM Tris HCl pH 8, six column volumes of 50 mM Tris HCl, pH 8. The MHC II molecules were eluted at room temperature for 4 min on a rotator by adding 1 ml of 10% acetic acid. Small aliquots (5 ml) of each elution fraction were analyzed by 10% SDS-PAGE and by Western blotting developed with an anti-mouse class II monoclonal antibody as culture supernatant from KL295 hybridoma([6](#_ENREF_6)) and a secondary anti-mouse IgG 1 -HRP conjugated antibody (SouthernBiotech) to evaluate the yield and purity of the eluted HLA.

## Purification of peptides bound to the MHC II molecules

MHC II peptide complexes were boiled at 70^o^C for 10 min. MHC II peptides were separated from the denatured protein subunits of the HLA molecules and the contaminating antibody by ultrafiltration through a 10 kD cutoff membrane filter (Sartorius Stedim, Aubagne, France) and centrifugation at 3000 g. Prior to the ultrafiltration, the filters were washed three times with 2 ml water to remove contaminants interfering with the mass spectrometry. Recovered peptide mixtures (5-6 ml) were concentrated and desalted with C-18 cartridge (Waters, Medford, MA). The C-18 cartridge was first washed three times with 50% acetonitrile (1.5 ml), equilibrated with 0.1% trifluoracetic acid (TFA) in water, and then loaded with the peptide mixture. The cartridge was then washed by an additional 3 ml 0.1 TFA%, and the peptides were eluted with 0.1% TFA in 50% acetonitrile in (1.5 ml). The eluted MHC peptides were reduced to near dryness and then reconstituted at 20 µL 0.1% TFA/water. Half of the peptide mixture, corresponding to approximately 3.5-4 x10^8^ cell equivalents, was injected for LC-MS/MS analysis.

## MS analysis

For LC-MS/MS analysis, the MHC peptide mixture was separated on the Dionex U3000 capillary/nano-HPLC system (Dionex, Sunnyvale, California) that is directly interfaced with the Thermo-Fisher LTQ-Obritrap mass spectrometer (Thermo Fisher, San Jose, California). Prior to the analysis by tandem LC-MS/MS, the complex mixture was passed through a 10 kD filter to separate the peptides bound to the MHC II complex from other higher molecular weight peptides and proteins that might be present in the mixture eluted from the Ab column as described above. The analytical column was a home-made fused silica capillary column (75 µm ID, 100 mm length; Upchurch, Oak Harbor, Washington) packed with C-18 resin (300 A, 5 mm, Varian, Palo Alto, California). To optimize the separation of peptides bound to the MHC II complex, the mixture was run on capillary/nano HPLC system with a shallow gradient of an aqueous mobile phase A (0.1% formic acid in water) and organic mobile phase B (0.1% formic acid in 100% acetonitrile) formed in 180 min with a flow rate of 250 nL/min under the following conditions: 0 % B to 55% B formed in 120 min, followed by 25 min gradient from 55% to 80% solvent B. Solvent B was maintained at 80% for another 10 min and then decreased to 0% in 10 min. Another 15 min interval was used for equilibration, loading and washing. The HPLC system was interfaced with the Thermo-Fisher LTQ Orbitrap XL mass spectrometer operated in the data-dependent acquisition mode using the Xcalibur 2.0.7 software. The experiment consisted of a single MS full-scan in the Orbitrap (620-1200 m/z, 30,000 resolution) followed by 6 data-dependent MS/MS scans in the ion trap at 35% normalized collision energy. The most intense 6 masses from each full mass spectrum with doubly and triply charge states were selected for fragmentation by collision-induced dissociation in the linear ion-trap. The dynamic exclusion parameters were as follows: Repeat count = 1; Repeat Duration = 30 second; Exclusion list = 100; and Exclusion time = 90 second.

The MS/MS spectra from each LC-MS/MS run were converted to mzXML using ProteoWizard. MS/MS spectra were inspected manually to confirm that the major fragmented ions matched the identified peptide sequences.

***EpiSifter.***

EpiSifter was implemented using Perl (www.perl.org) and R (www.r-project.org). First, sequences of the proteins of interest are cut to generate all possible MHC II peptides. For a protein of N amino acids, and the peptide length ranging from n_1_ to n_2_, and the number of charge states considered z, the number of possible mass to charge ratios is given by . If modifications are considered this number will increase, e.g. if methionine oxidation is considered, a peptide containing m methionines will generate as m/z ratios instead of . Second, the mass chromatograms for the possible peptides, peptide modifications, charge states, isotopic peaks, and interfering peaks are extracted from the mzXML files within the mass accuracy of the mass spectrometer (10 ppm). Third, the mass chromatograms are plotted together with the expected intensity ratio’s using R. These plots then allow the user to quickly assess the evidence for a large set of peptides. This semi-manual strategy utilizes the extraordinary human ability for pattern recognition, and therefore works well even for very low-intensity peaks for which most automatic methods are inconsistent. The candidate peptides that are selected are then targeted for MS/MS in a second experiment to confirm or reject them.

***Peptide Identification***

X! Tandem([7](#_ENREF_7), [8](#_ENREF_8)) (www.thegpm.org) was used for peptide identification using mass tolerances of 10 ppm and 0.4 Da for the precursor and fragment ions, respectively. Sequence collections for searching were constructed from the mouse proteome and HIV gag p24 protein and searched under the assumption that enzymatic cleavage can occur between any pair of amino acids. This will create a large search space with many irrelevant sequences that will not be present in the sample, and therefore decrease the sensitivity of the search. Therefore, a smaller search space focused on the possible MHC II peptides (7 to 30 amino acids long) from HIV gag p24 protein was also searched.

## Peptide synthesis

## Peptides were synthesized in collaboration with the Proteomics Resource Center, Rockefeller University, New York, NY. by the solid phase peptide synthesis method according to established protocols([9](#_ENREF_9)).

## For cell culture experiments, peptide libraries consisting of overlapping 15-mer peptides (staggered by 4 amino acids) spanning the entire HIV gag p24 and HIV gag p17 sequences, respectively, were synthesized on the robotic peptide synthesizer (Intavis) on a 10 μmol scale in 96-well plates as described([10](#_ENREF_10)). Synthetic peptides were stored as lyophilized powders at -20^o^C and dissolved in 100 % DMSO (1 mg/ml) prior to use. This stock solution was further diluted to desired concentrations and the concentrated stock solution stored at -80^o^C([10](#_ENREF_10)). Single peptides VDRFYKTLRAEQASQ (D6), DRFYKTLRAEQASQ (D6.1), QAISPRTLNAWVKVV (A4), PVGEIYKRWIILGLN (C8) and SPEVIPMFSALSEGA (A9) from the HIV gag p24 protein were synthesized on a SYMPHONY™ multiple peptide synthesizer (Protein Technologies, Tuscon, Arizona) on preloaded Wang resins (Bachem, Torrance, CA) on a 25 µmol scale, using Fmoc protected amino acids (Anaspec, Fremont, CA)([11](#_ENREF_11), [12](#_ENREF_12)).

For quantitation experiments by LC-MS analysis, stable isotopically labeled peptide were synthesized as reported([13](#_ENREF_13)) and purified to greater than 95% homogeneity. Briefly, Fmoc amino acids uniformly labeled with ^13^C and ^15^N to attain a greater molecular weight shift (Isotech, Miamisburg, OH) were introduced at appropriate positions (Supplemental Table 2). Peptides were resuspended at 1 mg/ml in 50% acetonitrile, 0.1% TFA in water prior their use in quantitative assays.

## Quantitation of MHC II peptides

An absolute quantitation method based on the combination of exact mass measurements, their retention time and the comparison of profile intensity of light peptides and isotopically labeled (heavy) peptides was used to quantitate selected MHC II peptides([14](#_ENREF_14), [15](#_ENREF_15)). Synthetic peptides for quantitation and their isotopically labeled counterparts are listed in Table S3. MHC II peptide mixture (10 µL) in 0.1 % TFA/water was mixed with 10 µL of a solution containing the two synthetic isotope labeled peptides dissolved in 50% acetonitrile/ 0.1% TFA/water. The amount of synthetic isotopically labeled peptides spiked into the sample was 1 ng. The entire peptide mixture was subjected for LC/MS analysis. Peptides were separated on the HPLC column under the same solvent gradient conditions as the ones described for the LC-MS/MS. The LTQ-Orbitrap mass spectrometer was operated in the full scan mode with a resolution of 30,000. In the LC–MS experiments we measured the peak intensity of a specific ion from both the native peptide and the isotopically labeled peptide as a function of m/z values. The absolute quantification was determined by comparing the peak intensity of the native peptide with the peak intensity of heavy peptide added at 1 ng. The actual copy numbers of MHC II-bound HIV gag p24 peptides from DCs were calculated as follows: 1) moles of native peptide determined by AQUA analysis = g/ MW; 2) Molecules of native peptide = moles (from step 1) x 6.022 x10^23^/mol; 3) Molecules of native peptide per cell = molecules (from step 2)/number of cells used per AQUA analysis.

## Expansion of HIV specific T cells in bulksplenocytes

To test peptide reactivity, we assessed proliferation of antigen primed T cells in presence of HIV gag p24 specific peptides. Antigen-specific bulk splenocytes were isolated from F1 mice primed intraperitoneally with 5 μg anti-DEC-p24 and 50 μg of poly ICLC, as adjuvant, and boosted with the same condition 4–6 weeks later [[20](#_ENREF_20)]. Spleens from immunized mice (2-4 mice) were collected 2 weeks later after boost. Bulksplenocytes isolated from HIV gag p24 primed F1 mice were labeled with CFSE (10^7^ cell/ml, 1 μM, 10 min at 37^0^C; Molecular Probes, Eugene, OR) and cultured in 96-well, round bottom plates with 2 μg/ml of anti-CD28 costimulatory antibody (clone 37.51) and either 0.05 μg/ml of HIV gag p24 peptide mix, HIV gag p24 ***VDRFYKTLRAEQASQ (D6)*** and ***DRFYKTLRAEQASQ (D6.1)*** peptides, HIV gag p17 as negative control or ApoE peptide (KELEEQLGPVAEETR) as non-reactive peptide([13](#_ENREF_13)). After 3 days of culture, samples were restimulated for 6 h with the corresponding peptide (2 μg/ml) and anti-CD28, adding BFA at 10 μg/ml for the last 5 h to allow accumulation of intracellular cytokines. Cells were washed, incubated for 5 min at 4^0^C with 2.4G2 mAb to block Fcγ receptors, washed, and stained with Live/Dead Fixable Aqua viability dye (Invitrogen, Carlsbad, CA), Alexa Fluor 700 conjugated anti-CD3, PerCP-Cy 5.5 conjugated anti-CD4, APC Alexa 780 conjugated anti-CD8 (eBiosciences, San Diego, CA) mAbs for 20 min at 4^0^C. Cells were permeabilized (Cytofix/Cytoperm Plus; BD Biosciences) and stained with APC-conjugated anti-IFNγ mAb (BD Biosciences) for 15 min at RT, washed and resuspended in PBS and live-CD3^+^ cells (50,000) were acquired on a BD LSR II flowcytometer. Data were analyzed with FlowJo Software (Tree Star, Inc., San Carlos, CA).

## Expansion of HIV specific T cells in DC: T cell coculture.

To directly assess the function of DCs in presenting HIV gag p24 peptides, we used in parallel to the bulksplenocyte assay above, Flt3L-mobilized splenic DCs. A total of 2x10^6^ splenic CD11c^+^ DC were first pulsed with different HIV gag p24 peptide concentrations for 5 h in 24-well culture plates in a final volume of 0.5 mL of complete medium. Then, 25 μg/mL poly IC was added to the culture. In some experiments, NH_4_Cl (20 mM) and chloroquine (100 μM) (Sigma, St. Louis, Mo) were used to pre-treat a fraction DCs. Treated or untreated DCs were loaded with peptides for 5 h, washed three times with PBS and added to CFSE labeled HIV gag p24 -primed T cells at a ratio of 1:20. The cocultures were incubated at 37^0^C for 4 days. Each sample was then restimulated for 6 h with the corresponding HIV gag p24 peptide (2 μg/ml) in the presence of costimulatory anti-CD28 mAb (2 μg/ml). IFN-γ secretion and cell proliferation by CFSE dilution was monitored as described above.

***Functional avidity of MHC II-bound HIV gag p24 peptides in DC:T cell coculture***

To assess functional avidity of the peptide-specific T cells responses, the magnitude of the CD4+ T cell response at each concentration of peptide measured as % of IFNg^+^ CFSE^low^ cells was expressed as percentage of the of the maximum response observed after stimulation with 1x10^-6^ M peptide. We extrapolated the concentration at which 50% of the maximal response was reached by using nonlinear regression within Prism 3 program (Graphpad Software, La Jolla, CA).

**References**

1. Dranoff G*, et al.* (1993) Vaccination with irradiated tumor cells engineered to secrete murine granulocyte-macrophage colony-stimulating factor stimulates potent, specific, and long-lasting anti-tumor immunity. *Proc. Natl. Acad. Sci. USA* 90:3539-3543.

2. Pulendran B*, et al.* (1997) Developmental pathways of dendritic cells in vivo: distinct function, phenotype, and localization of dendritic cell subsets in FLT3 ligand- treated mice. *J. Immunol.* 159:2222-2231.

3. Bozzacco L*, et al.* (2010) HIV gag protein is efficiently cross-presented when targeted with an antibody towards the DEC-205 receptor in Flt3 ligand-mobilized murine DC. (Translated from eng) *Eur. J. Immunol.* 40(1):36-46 (in eng).

4. Metlay JP*, et al.* (1990) The distinct leukocyte integrins of mouse spleen dendritic cells as identified with new hamster monoclonal antibodies. *J. Exp. Med.* 171:1753-1771.

5. Trumpfheller C*, et al.* (2008) The microbial mimic poly IC induces durable and protective CD4^+^ T cell immunity together with a dendritic cell targeted vaccine. *Proc. Natl. Acad. Sci. USA* 105:2574-2579.

6. Kang Y-S*, et al.* (2003) SIGN-R1, a novel C-type lectin expressed by marginal zone macrophages in spleen, mediates uptake of the polysaccharide dextran. *Int. Immunol.* 15:177-186.

7. Craig R & Beavis RC (2004) TANDEM: matching proteins with tandem mass spectra. (Translated from eng) *Bioinformatics* 20(9):1466-1467 (in eng).

8. Fenyo D, Eriksson J, & Beavis R (2010) Mass spectrometric protein identification using the global proteome machine. (Translated from eng) *Methods Mol. Biol.* 673:189-202 (in eng).

9. Sole NA & Barany G (1992) Optimization of solid-phase synthesis of [Ala^8^]-dynorphin A. *J. Organic Chem.* 57(20):5399-5403.

10. Trumpfheller C*, et al.* (2006) Intensified and protective CD4^+^ T cell immunity in mice with anti-dendritic cell HIV gag fusion antibody vaccine. *J. Exp. Med.* 203:607-617.

11. Wellings DA & Atherton E (1997) Standard Fmoc protocols. (Translated from eng) *Methods Enzymol.* 289:44-67 (in eng).

12. Knorr R, Trzeciak A, Bannwarth W, & Gillessen D (1989) New coupling reagents in peptide chemistry. *Tetrahedron Lett.* 30(15):1927-1930.

13. Bozzacco L*, et al.* (2011) Mass spectrometry analysis and quantitation of peptides presented on the MHC II molecules of mouse spleen dendritic cells. (Translated from eng) *J. Proteome Res.* 10(11):5016-5030 (in eng).

14. Gerber SA, Rush J, Stemman O, Kirschner MW, & Gygi SP (2003) Absolute quantification of proteins and phosphoproteins from cell lysates by tandem MS. (Translated from eng) *Proc. Natl. Acad. Sci. USA* 100(12):6940-6945 (in eng).

15. Hardt M, Witkowska HE, Hall SC, & Fisher S (2008) Absolute quantitation of targeted endogenous salivary peptides using heavy isotope-labeled internal standards and high-resolution selective reaction monitoring mass spectrometry. *Thermo Scientific* Application Note: 451.
